# Supplementary material for: Action subsampling supports policy compression in large action spaces
Source: PLoS Comput Biol. 2025 Sep 5;21(9):e1013444. doi: 10.1371/journal.pcbi.1013444 (PMC12422588; doi:10.1371/journal.pcbi.1013444)
Supplement: S1 Appendix — (PDF) [file pcbi.1013444.s001.pdf]

Action subsampling supports policy compression in large action spaces

Shuze Liu[1*], Samuel Joseph Gershman[2,3]

1 PhD Program in Neuroscience, Harvard University, Cambridge, Massachusetts, USA
2 Department of Psychology and Center for Brain Science, Harvard University, Cambridge, Massachusetts, USA
3 Center for Brains, Minds, and Machines, Massachusetts Institute of Technology, Cambridge, Massachusetts, USA

* shuzeliu@fas.harvard.edu

S1 Appendix

Table A. Human behavioral results, using action counting threshold of $\epsilon = 1$. The number of actions N_a counts every distinct action that has been taken by the participant. For LMEs, fixed effects and their p-values are reported; for Pearson correlations, the coefficients and their p-values are reported. The first column corresponds to all included participants (reported in the main text), while the second column analyzes only the subgroup of included participants with trial-averaged reward > 0.15 for every test block.

	$\epsilon = 1$ No reward cutoff	$\epsilon = 1$ Reward > 0.15 cutoff
LME 1 RTDeadlineCond	0.762 ± 0.0778 ($p < 10^{-18}$)	0.842 ± 0.0859 ($p < 10^{-17}$)
LME 2 RTDeadlineCond	0.249 ± 0.0265 ($p < 10^{-17}$)	0.265 ± 0.0285 ($p < 10^{-16}$)
LME 3 RTDeadlineCond	-0.309 ± 0.0325 ($p < 10^{-17}$)	-0.358 ± 0.0384 ($p < 10^{-16}$)
LME 4 PolicyComplexity	0.273 ± 0.0103, ($p < 10^{-69}$)	0.267 ± 0.00895 ($p < 10^{-71}$)
LME 5 RTDeadlineCond	1.30 ± 0.159 ($p < 10^{-13}$)	1.53 ± 0.182 ($p < 10^{-14}$)
LME 6 Na	0.0861 ± 0.00671 ($p < 10^{-27}$)	0.102 ± 0.00553 ($p < 10^{-43}$)
LME 7 PolicyComplexity	0.248 ± 0.0209 ($p < 10^{-24}$)	0.230 ± 0.0216 ($p < 10^{-20}$)
LME 7 PolicyComplexity : Na	0.0205 ± 0.00306 ($p < 10^{-10}$)	0.0185 ± 0.00324 ($p < 10^{-7}$)
LME 8 PolicyComplexity	-0.276 ± 0.0223 ($p < 10^{-26}$)	-0.272 ± 0.0350 ($p < 10^{-12}$)
LME 8 PolicyComplexity : Na	0.0571 ± 0.00341 ($p < 10^{-40}$)	0.0514 ± 0.00545 ($p < 10^{-16}$)
Correlation 9	$R = 0.671$ ($p < 10^{-30}$)	$R = 0.828$ ($p < 10^{-47}$)
Correlation 10	$R = 0.792$ ($p < 10^{-16}$)	$R = 0.766$ ($p < 10^{-12}$)
Correlation 11	$R = 0.327$ ($p = 0.00416$)	$R = 0.907$ ($p < 10^{-4}$)
LME 12 PolicyComplexity	0.347 ± 0.0498 ($p < 10^{-10}$)	0.762 ± 0.0778 ($p < 10^{-18}$)
LME 12 Na	0.0146 ± 0.00625 ($p = 0.0200$)	-0.0259 ± 0.00786 ($p < 0.01$)

Table B. Human behavioral results, using action counting threshold of $\epsilon = 2$. The number of actions N_a counts every distinct action that has been taken by the participant at least twice. For LMEs, fixed effects and their p-values are reported; for Pearson correlations, the coefficients and their p-values are reported. Red denotes effects that do not significantly differ from 0 under the $\alpha = 0.05$ cutoff.

	$\epsilon = 2$ No reward cutoff	$\epsilon = 2$ Reward > 0.15 cutoff
LME 1 RTDeadlineCond	0.762 ± 0.0778 ($p < 10^{-18}$)	0.842 ± 0.0859 ($p < 10^{-17}$)
LME 2 RTDeadlineCond	0.249 ± 0.0265 ($p < 10^{-17}$)	0.265 ± 0.0285 ($p < 10^{-16}$)
LME 3 RTDeadlineCond	-0.309 ± 0.0325 ($p < 10^{-17}$)	-0.358 ± 0.0384 ($p < 10^{-16}$)
LME 4 PolicyComplexity	0.273 ± 0.0104, ($p < 10^{-69}$)	0.267 ± 0.00895 ($p < 10^{-71}$)
LME 5 RTDeadlineCond	1.32 ± 0.162 ($p < 10^{-13}$)	1.58 ± 0.189 ($p < 10^{-13}$)
LME 6 Na	0.0937 ± 0.00649 ($p < 10^{-33}$)	0.110 ± 0.00468 ($p < 10^{-56}$)
LME 7 PolicyComplexity	0.233 ± 0.0253 ($p < 10^{-16}$)	0.183 ± 0.0242 ($p < 10^{-11}$)
LME 7 PolicyComplexity : Na	0.0239 ± 0.00345 ($p < 10^{-10}$)	0.0274 ± 0.00359 ($p < 10^{-11}$)
LME 8 PolicyComplexity	-0.276 ± 0.0223 ($p < 10^{-26}$)	-0.312 ± 0.0287 ($p < 10^{-20}$)
LME 8 PolicyComplexity : Na	0.0596 ± 0.00427 ($p < 10^{-31}$)	0.0608 ± 0.00431 ($p < 10^{-30}$)
Correlation 9	$R = 0.727$ ($p < 10^{-37}$)	$R = 0.895$ ($p < 10^{-65}$)
Correlation 10	$R = 0.792$ ($p < 10^{-16}$)	$R = 0.766$ ($p < 10^{-12}$)
Correlation 11	$R = 0.369$ ($p = 0.00113$)	$R = 0.589$ ($p < 10^{-6}$)
LME 12 PolicyComplexity	0.327 ± 0.0482 ($p < 10^{-9}$)	0.762 ± 0.0778 ($p < 10^{-18}$)
LME 12 Na	0.0112 ± 0.00667 ($p = 0.0957$)	0.0487 ± 0.00963 ($p < 10^{-5}$)

Table C. Human behavioral results, using action counting threshold of $\epsilon = 3$. The number of actions N_a counts every distinct action that has been taken by the participant at least three times. For LMEs, fixed effects and their p-values are reported; for Pearson correlations, the coefficients and their p-values are reported. Red denotes effects that do not significantly differ from 0 under the $\alpha = 0.05$ cutoff.

	$\epsilon = 3$<br>No reward cutoff	$\epsilon = 3$<br>Reward > 0.15 cutoff
LME 1<br>RTDeadlineCond	0.762 ± 0.0778 ($p < 10^{-18}$)	0.842 ± 0.0859 ($p < 10^{-17}$)
LME 2<br>RTDeadlineCond	0.249 ± 0.0265 ($p < 10^{-17}$)	0.265 ± 0.0285 ($p < 10^{-16}$)
LME 3<br>RTDeadlineCond	-0.309 ± 0.0325 ($p < 10^{-17}$)	-0.358 ± 0.0384 ($p < 10^{-16}$)
LME 4<br>PolicyComplexity	0.273 ± 0.0104, ($p < 10^{-69}$)	0.267 ± 0.00895 ($p < 10^{-71}$)
LME 5<br>RTDeadlineCond	1.40 ± 0.167 ($p < 10^{-14}$)	1.67 ± 0.197 ($p < 10^{-14}$)
LME 6<br>Na	0.0975 ± 0.00605 ($p < 10^{-38}$)	0.113 ± 0.00390 ($p < 10^{-69}$)
LME 7<br>PolicyComplexity	0.229 ± 0.0246 ($p < 10^{-16}$)	0.158 ± 0.0248 ($p < 10^{-9}$)
LME 7<br>PolicyComplexity : Na	0.0259 ± 0.00342 ($p < 10^{-11}$)	0.0316 ± 0.00367 ($p < 10^{-14}$)
LME 8<br>PolicyComplexity	-0.253 ± 0.0281 ($p < 10^{-16}$)	-0.278 ± 0.0337 ($p < 10^{-13}$)
LME 8<br>PolicyComplexity : Na	0.0603 ± 0.00418 ($p < 10^{-32}$)	0.0603 ± 0.00486 ($p < 10^{-25}$)
Correlation 9	$R = 0.765$ ($p < 10^{-43}$)	$R = 0.931$ ($p < 10^{-81}$)
Correlation 10	$R = 0.792$ ($p < 10-16^)$	$R = 0.766$ ($p < 10^{-12}$)
Correlation 11	$R = 0.417$ ($p < 10^{-3}$)	$R = 0.648$ ($p < 10^{-7}$)
LME 12<br>PolicyComplexity	0.433 ± 0.0556 ($p < 10^{-12}$)	0.762 ± 0.0778 ($p < 10^{-18}$)
LME 12<br>Na	0.0147 ± 0.00766 ($p = 0.0561$)	0.0487 ± 0.00963 ($p < 10^{-5}$)
