# Supplementary figures and images for: Action subsampling supports policy compression in large action spaces
Source: PLoS Comput Biol. 2025 Sep 5;21(9):e1013444. doi: 10.1371/journal.pcbi.1013444 (PMC12422588; doi:10.1371/journal.pcbi.1013444)

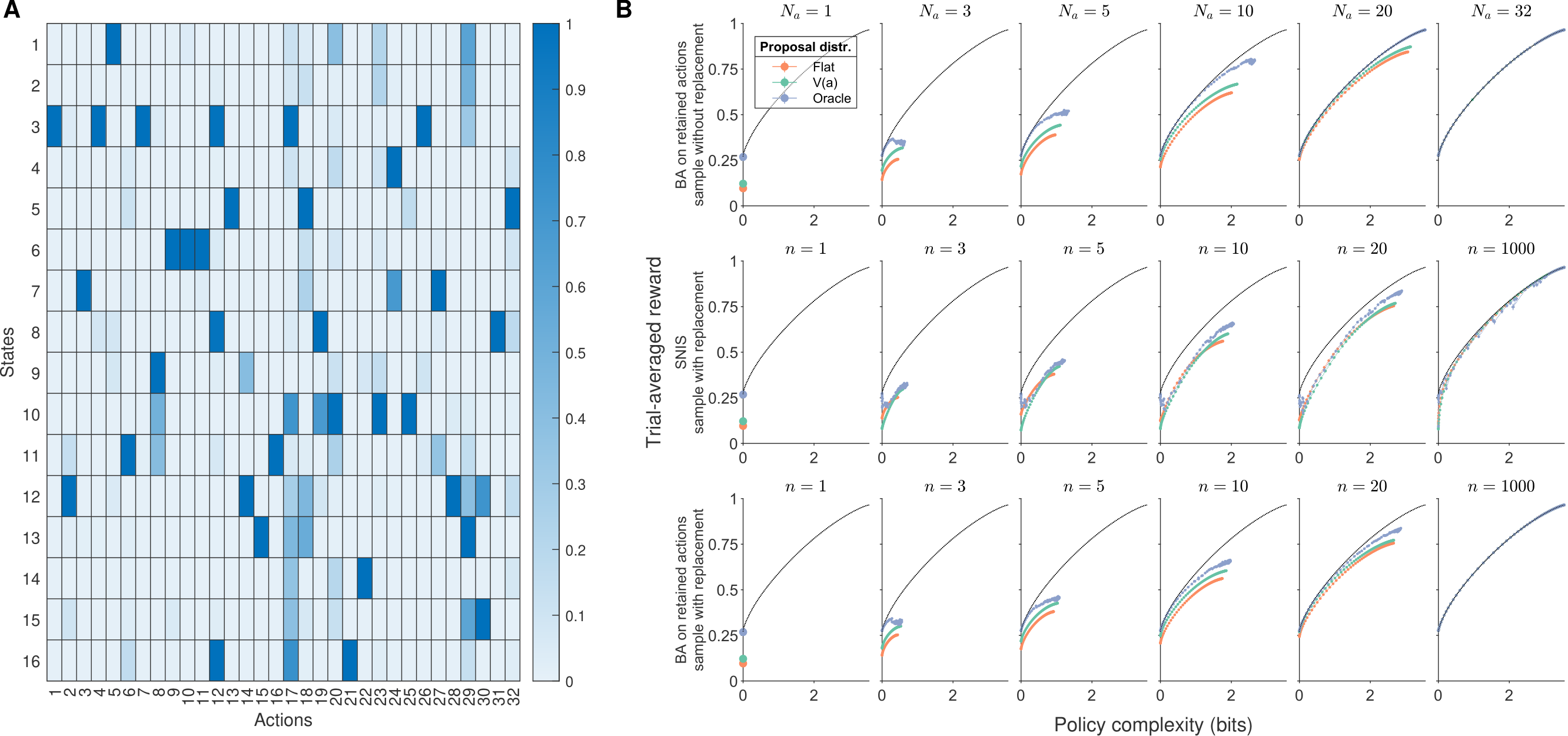

Supplement: S1 Fig — Rows structure is identical to Fig 3. Columns denote trial-averaged reward instead of loss in trial-averaged reward. (TIFF) [file pcbi.1013444.s002.tiff]

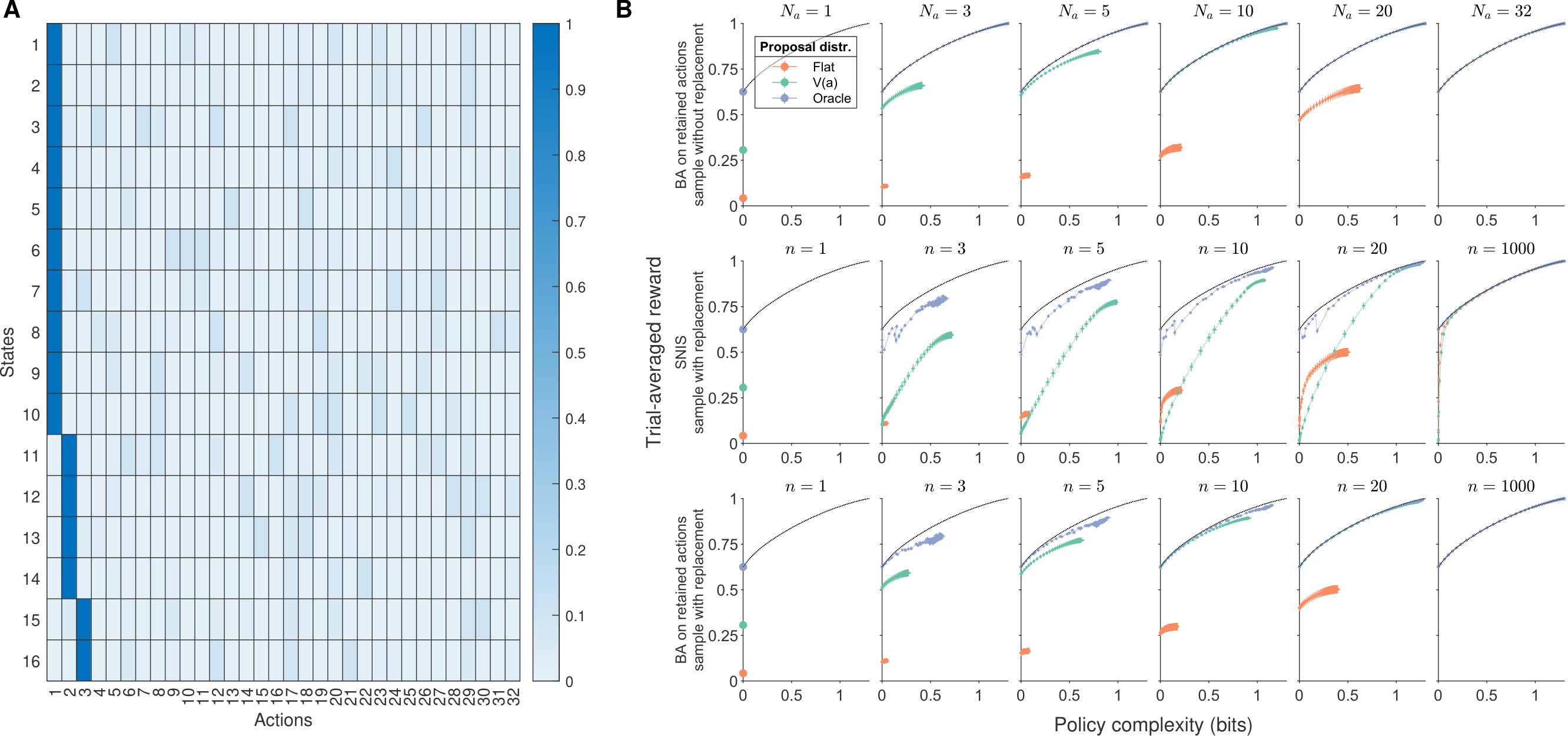

Supplement: S2 Fig — Row structure is identical to S1 Fig. (TIFF) [file pcbi.1013444.s003.tiff]

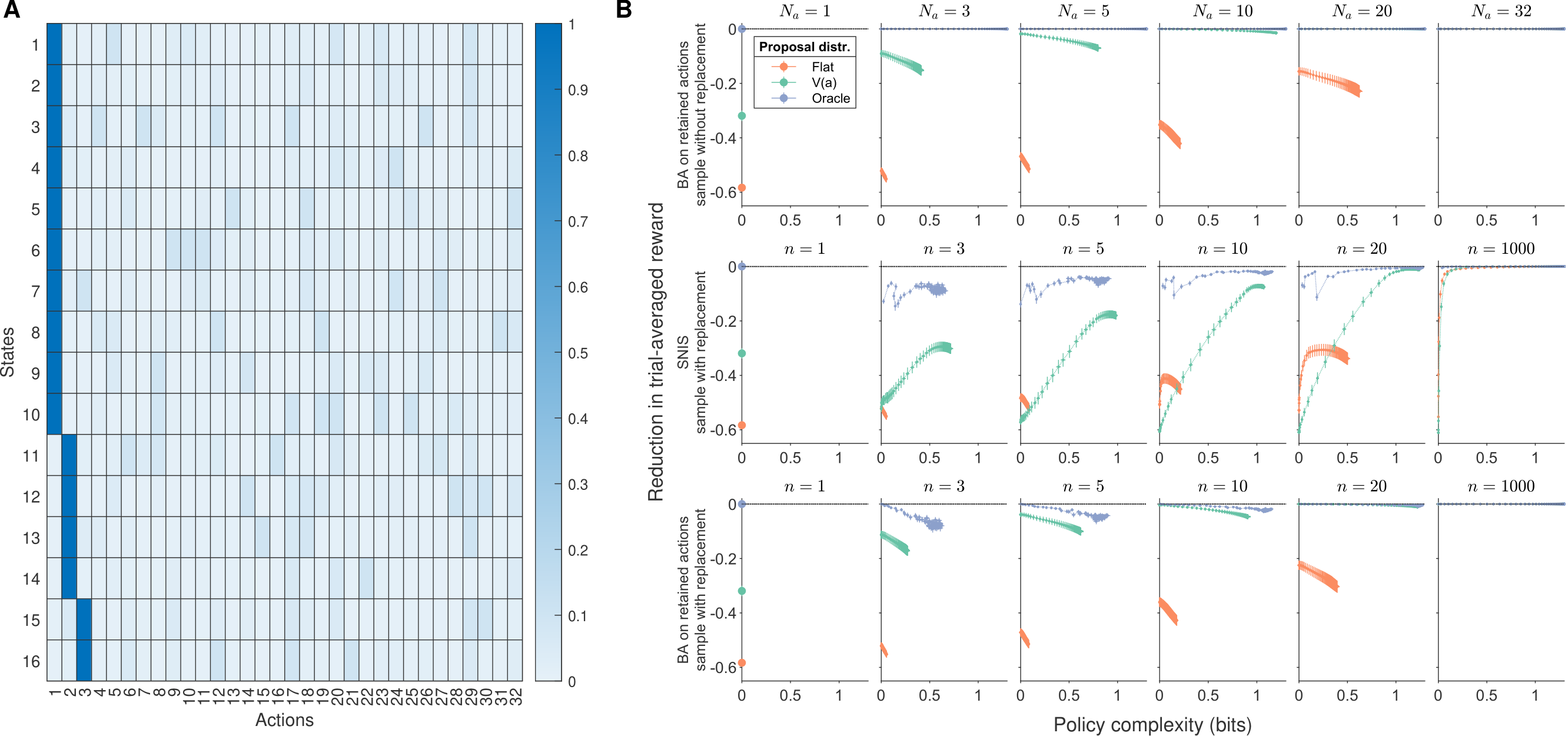

Supplement: S3 Fig — Row and column structure are identical to Fig 3. (TIFF) [file pcbi.1013444.s004.tiff]

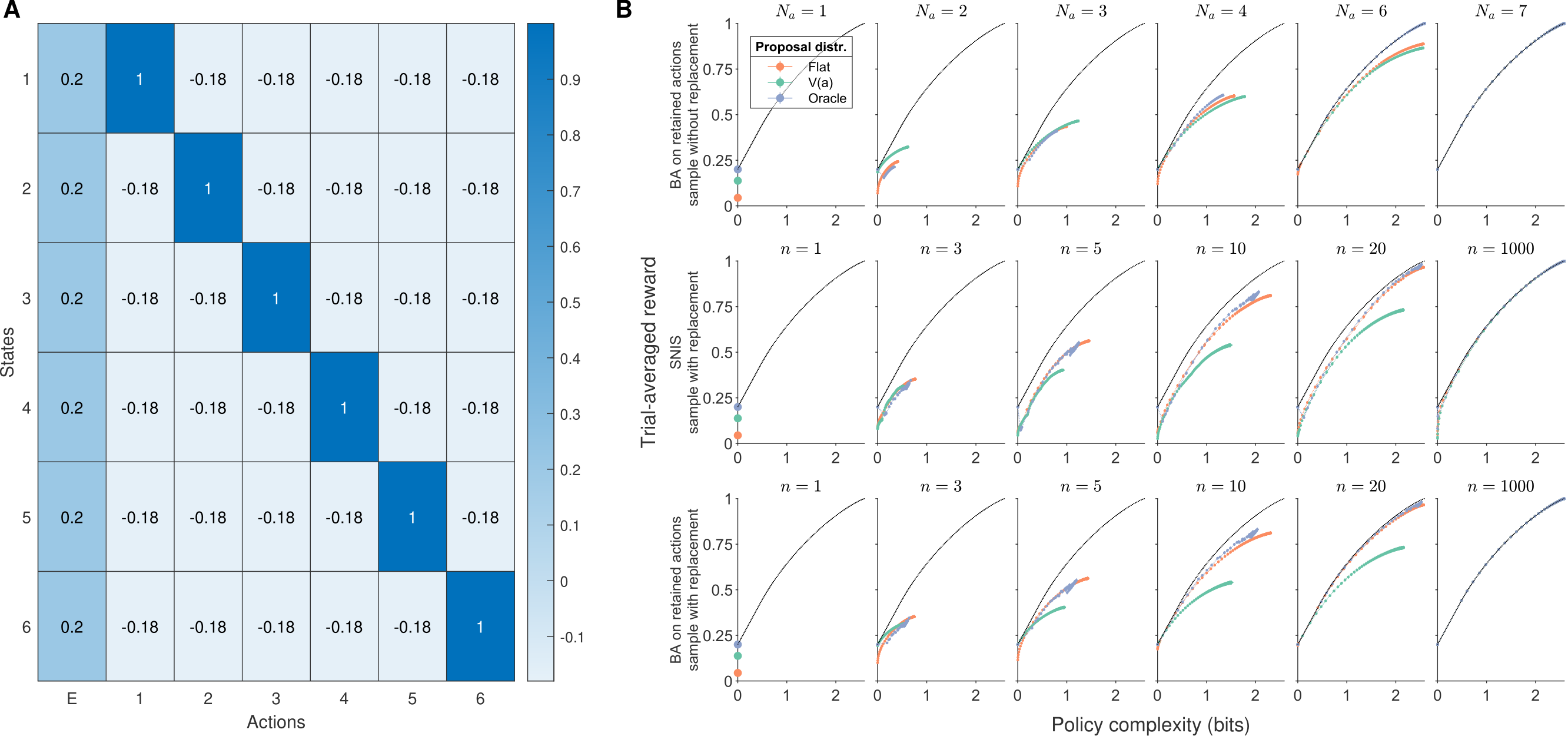

Supplement: S4 Fig — Rows and column structures are identical to S1 Fig. (TIFF) [file pcbi.1013444.s005.tiff]

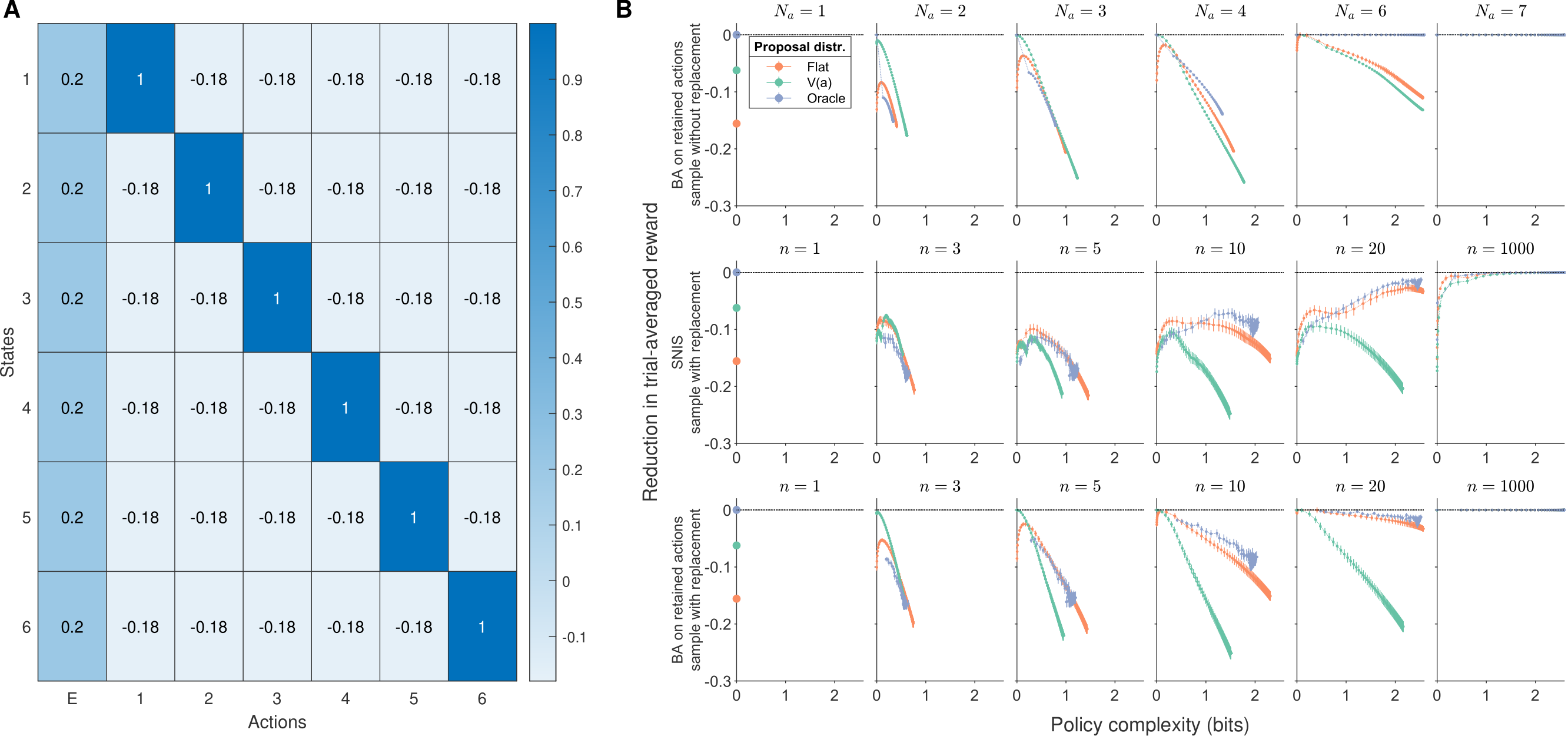

Supplement: S5 Fig — Rows and column structures are identical to Fig 3. (TIFF) [file pcbi.1013444.s006.tiff]

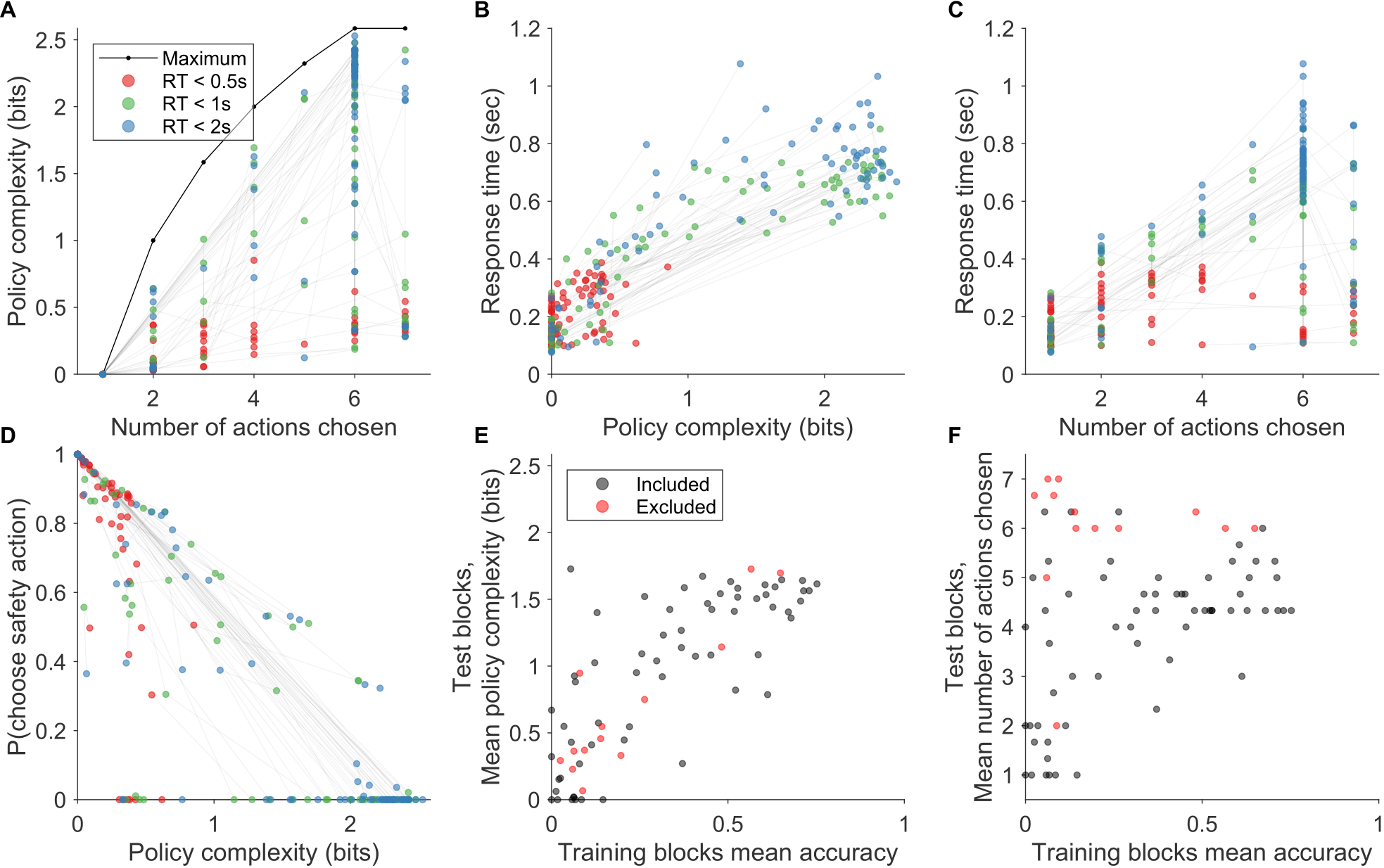

Supplement: S6 Fig — Relationships between (A) number of actions chosen and policy complexity (black solid line denotes maximum policy complexity enabled by each number of actions chosen), (B) policy complexity and RT, (C) number of actions chosen and RT, (D) policy complexity and probability of choosing the safety action. Color denotes the RT deadline condition, and semitransparent gray lines connect the same participant’s data. Training and test block relationships include (E) training block mean accuracy and test block mean policy complexity, and (F) training block mean accuracy and test block mean number of actions chosen. Color denotes the participants we have included or excluded based on the reward >0.15 cutoff. (TIFF) [file pcbi.1013444.s007.tiff]

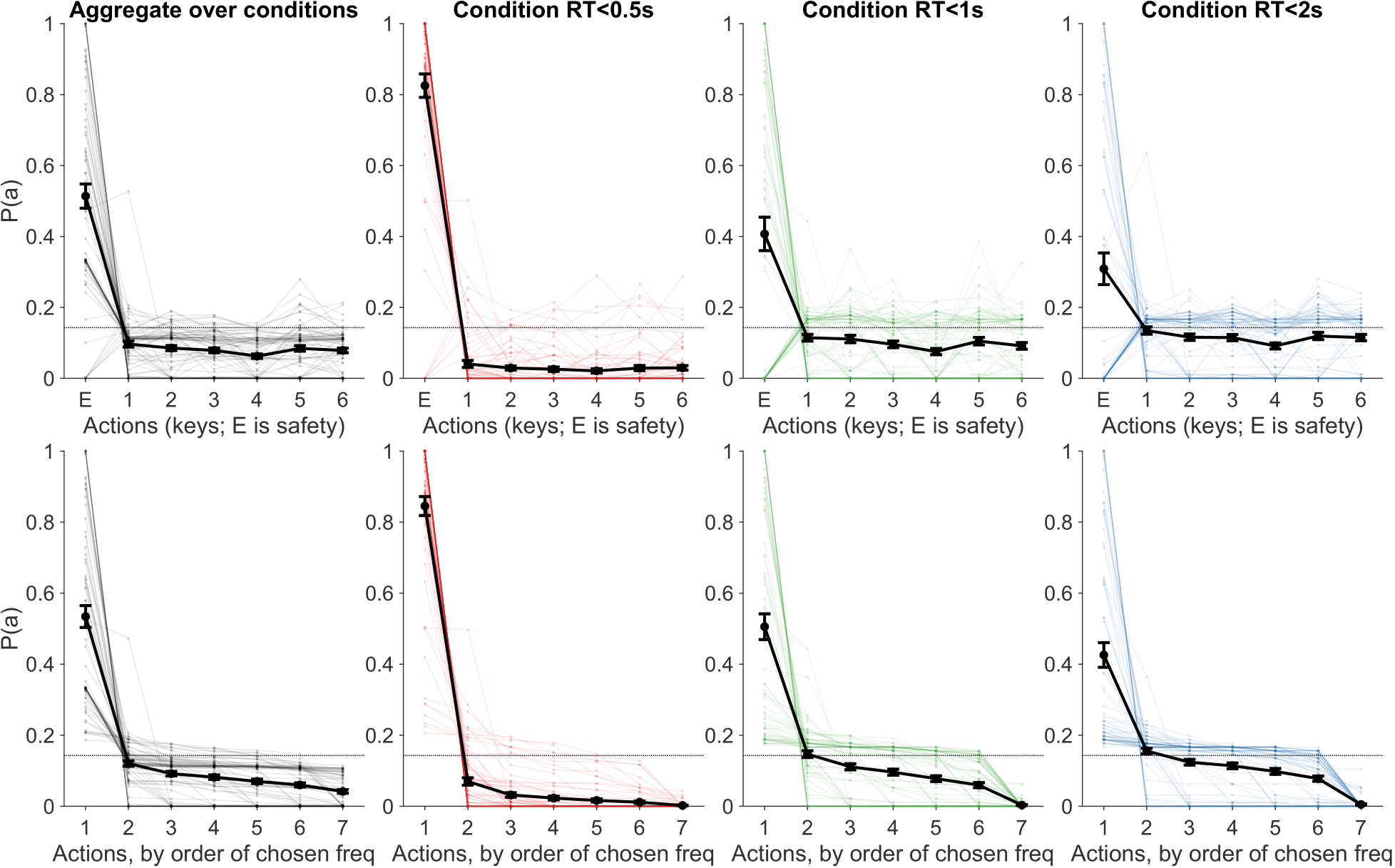

Supplement: S8 Fig — Row 1: Each participant’s empirical action distribution P(a) over all action keys (semitransparent lines; color denotes RT deadline condition), as well as their mean ± SEM (black errorbars). Row 2: Same as Row 1, but the actions are reordered for each participant based on their relative frequency. The black horizontal dotted line denotes chance probability of 1/7. (TIFF) [file pcbi.1013444.s009.tiff]

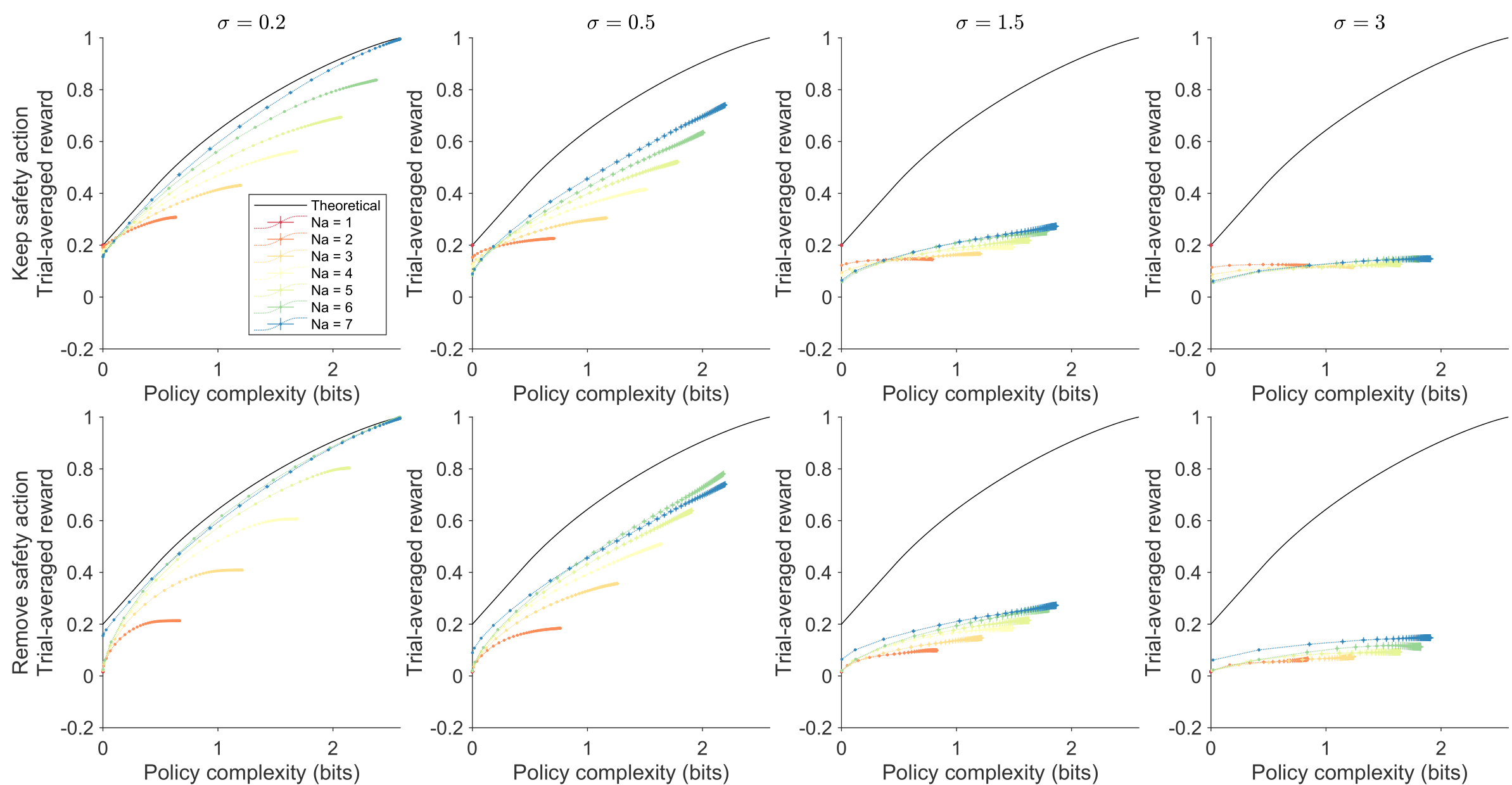

Supplement: S9 Fig — The human experiment task’s Q(s,a) values are smeared with Gaussian noise with increasing standard deviation (stratified into columns). Row 1: Assuming that the safety action is retained in the consideration set, the influence of policy complexity (x-axis) and action consideration set size (color) on trial-averaged reward (y-axis). The full-action-space reward-complexity frontier is depicted as a black line. 2D errorbars denote mean±SEM of policy complexity and trial-averaged reward over 200 random simulations, aggregating over simulations that share the same β. Row 2: Same as Row 1, but Assuming that the safety action is not retained in the consideration set. (TIFF) [file pcbi.1013444.s010.tiff]
