# Supplementary material for: Action subsampling supports policy compression in large action spaces
Source: PLoS Comput Biol. 2025 Sep 5;21(9):e1013444. doi: 10.1371/journal.pcbi.1013444 (PMC12422588; doi:10.1371/journal.pcbi.1013444)
Supplement: Attachment — Submitted filename: response_to_reviewers.pdf [file pcbi.1013444.s011.pdf]

Dear Dr. Doumas, Dr. Martin, and Reviewers,

Thank you very much for a highly constructive peer review. Both Reviewers found our theoretical framework interesting and provided valuable suggestions to improve logical clarity and strengthen the manuscript's motivation. We have carefully addressed these comments, and the revised manuscript is significantly improved as a result. Our responses to each point are provided in a detailed comment-by-comment reply. Below, we summarize the main revisions.

1. Introduction
   - Rephrased a previously misleading sentence that implied a dichotomy between meta-reasoning and resource constraints, instead emphasizing their potential concurrence.
2. Experiments and Analyses
   - Defined mathematical notations at their first appearance for clarity.
   - Clarified that the framework does not require the use of ground-truth $Q(s, a)$ values and can instead rely on relevant estimates.
   - Explicitly stated our hypothesis that general-value-based action sampling serves as an ecologically rational heuristic that generalizes well across tasks.
   - Emphasized the value of general-value-based sampling, given that identifying the optimal action consideration set for every task and policy complexity level is computationally intractable for agents and difficult to generalize.
   - Improved the presentation of bias-correction algorithms and explained why sampling actions with replacement provides better opportunities for bias correction.
   - Provided additional background on the motivation for studying self-normalized importance sampling (SNIS) and contrasted its objective with that of the Blahut-Arimoto (BA) algorithm.
   - Clarified the interpretation of simulation results.
   - Justified the use of correlation measures in analyzing human behavior.
3. Figures
   - Replaced stimulus images with similar ones made by the same authors, but under the CC BY 4.0 license (Figure 4A,C).
4. Discussion
   - Re-emphasized the role of general-value-based action sampling as a heuristic that generalizes across tasks.
   - Restated the study's primary focus on the synergistic interaction between policy complexity and action consideration sets in mitigating suboptimalities.
   - Explicitly mentioned the possibility that action consideration set construction and policy optimization may compete for the same cognitive resource pool, suggesting this as a direction for future research.
   - Expanded the discussion on suboptimal patterns observed at intermediate policy complexity levels.
5. Methods
   - Confirmed that all participants have given written consent.
   - Explained how the Blahut-Arimoto algorithm can be extended to continuous action spaces.
   - Compared and contrasted policy compression with other reinforcement learning (RL) paradigms.
   - Provided additional details on policy complexity estimation.

Editors:

Overall summary

Thank you for submitting your manuscript to PLOS Computational Biology. We have received two reviews from experts in the field. I am in full agreement with both reviewers that the paper is interesting, well motivated, and ambitious. I also, like reviewer 2, think that this paper would have a wide appeal within the cognitive science community. I think the both reviewers raise reasonable points, and I won't repeat them all here, but I think addressing these points will make for a much stronger paper. I will, however, emphasise the points of reviewer 2 that the writing in this paper could be made much clearer. I would advise thinking about reducing jargon, reducing repeated information across sections, and using consistent wording.

We thank the Editors for summarizing these common themes, and hope to address the underlying issues.

Minor comments

Reviewer 2 gives several such examples, and I'll provide a few of my own: On page 2 of the ms., there is a reference to Fig.1A. However, the figure includes several terms that are defined later in the text. When I first read the paper I spend a couple of minutes trying to figure out what I had missed. I think it would be much clearer to just say, "These findings have inspired a descriptive... state-dependent action evaluation, which we present below".

As the two-stage framework was developed prior to and independently of our current work, we feel that referencing its corresponding panel (Fig 1A) after introducing our framework's mathematical notation may confuse readers further. To balance narrative coherence with notation clarity, we have instead enhanced the figure captions for Fig 1 to provide a detailed description of the relevant mathematical notations:

> When facing a set of possible actions $\{a_1, ..., a_N\}$, agents first sample an action consideration set, where each action's probability of being sampled is proportional to its general value $V(a_i)$ across all contexts/states $\{s_1, ..., s_M\}$. Then, given the current context s, one action is chosen by comparing the context-specific value of all actions retained in the consideration set, $Q(s, a_i)$.

The term "general-value-based action" is used on line 29 before it is defined. On lines 201, and 211 the terms "general-value-guided action sampling" and "general-value-based sampling" are used. Are these meant to be different concepts? If not, why use different terms?

We thank the Editors for pointing out this. The two terms refer to the same concept, and we have changed all of them to "general-value-based" to ensure consistency. We now also introduce the concept formally when it first appears:

> Regarding action consideration sets, evidence suggests that humans tend to generate a small number of actions that have high general value across various contexts (i.e., general-value-based action sampling; Klein et al. (1995); Raab et al. (2009); Leder et al. (2018); Kaiser et al. (2013); Del Missier et al. (2015); Morris et al. (2021)).

On line 92, an equation is presented, and the term $Q(s, a)$ is not defined until line 117, after another equation using the term is presented, and in a completely different paragraph.

We now explain the mathematical notions when they first appear in the manuscript:

> Consequently, for a given task with state probability distribution $P(s)$ and state-specific action rewards $Q(s, a)$, agents would maximize trial-averaged reward $V^\pi = \sum_s P(s) \sum_a \pi(a|s)\ Q(s, a)$ under their constraint $I^\pi(S; A) \leq C$.

Again, I emphasise that it is only because I think this paper would be of interest to a wider audience that I pick these nits. I hope that these and the reviewer comments help you to revise your ms.

We thank the Editors for suggesting these changes, which have made our manuscript much more readable!

Reviewer 1:

Overall summary

Overall the paper is well structured in terms of introducing the main ideas of the paper, presenting simulations that motivate these ideas, and concluding the human subject experiments to make concrete determinations related to the questions under consideration. However in terms of the overall motive there are some things that are less clear, this is the majority of the comments on specific lines that I have raised below. Many of the points below are clarifications and if they can be resolved in an author reply then they need not necessarily be added to the manuscript itself.

We appreciate the Reviewer's generous comments. We hope to address these concerns to make our manuscript better.

Minor comments

I think that the background and theoretical motivation could benefit from a discussion of the similarities and differences between this proposed method and information-constraints in continuous action spaces in RL. Another connection that I would like to be clarified is between selecting action consideration sets and MARL settings where one agent's action follows another agent, this is especially relevant in settings where there is an information asymmetry between agents. If these two areas of research are not relevant then a short description of them and why they are not related would benefit the manuscript.

We appreciate the Reviewer's suggestions and the opportunity to clarify these potential connections.

Regarding parallels with reinforcement learning (RL), the proposed policy compression framework does share similarities with KL-regularized control in terms of optimal policy expressions (Todorov, 2009). We now expand on their similarity and differences in the Methods section:

> The optimal policy derived from policy compression shares similarities with the policy prescribed by Kullback-Leibler (KL) regularized control (Todorov, 2009), but there are key differences in how each framework handles action distributions and optimization constraints.
>
> In KL-regularized control, a predefined default action distribution—typically uniform over actions—is assumed. Deviations from this distribution are penalized as a cost (negative reward), with β in Eq 2 interpreted as a cost sensitivity parameter. In contrast, our framework replaces this fixed distribution with a flexible marginal action distribution, $P^*(a)$, and formulates the problem as a constrained optimization with $I^\pi(S;A) \leq C$, where β acts as a Lagrange multiplier.
>
> A distinguishing feature of policy compression is the dependence of $P^*(a)$ on the policy $\pi^*(a|s)$. This property is central to its normative implications and behavioral predictions. Specifically, it predicts a tendency toward perseveration, where agents favor actions they have frequently chosen in the past—especially when policy complexity is low (β is small), making $P^*(a)$ the dominant term in Eq 2. In contrast, KL-regularized control enforces policy regularization toward a fixed default distribution that is independent of the policy, not optimized for individual tasks, and unable to account for perseverative behavior that exploits task regularities.

Regarding generalizations to continuous action spaces, Equation 2 remains applicable if the action space is appropriately discretized. We now also include relevant information in the Methods:

> While the BA algorithm is formally defined for discrete state and action spaces, it can be naturally extended to continuous spaces through appropriate discretization. However, since our study focuses on sampling actions into consideration sets, the resulting action consideration set remains discrete by definition, avoiding the need for discretization. Future work could extend the framework to continuous action spaces through adaptive discretization techniques and investigate corresponding patterns in human behavior.

Finally, in response to the Reviewer's inquiry about multi-agent RL applications, our framework addresses the internal cognitive constraints of individual agents and how such constraints affect their decision making. We believe that multi-agent RL applications, while quite interesting, are outside the scope of this paper.

Lines 49-50: An agent that is able to deliberately meta-reason about how best to construct consideration sets seems to necessarily have either a higher capacity or more time to consider how best to construct the consideration set before selecting an action. This is highly distinct from the case where an agent is compelled by resource limitations to simplify the rate-distortion problem over a reduced consideration subset. It is unclear from the introduction whether the proposed method is intended to handle one of these specific cases or both.

We appreciate the Reviewer's insightful comment and recognize the need for greater clarity in our framing. While we acknowledge that meta-reasoning about consideration sets can require additional cognitive resources or time, we do not see a fundamental distinction between the two cases raised. Both scenarios allow for individual agency in determining not only the number of actions considered but also which specific actions to include. Even under strict resource limitations (e.g., time constraints in our experiment), agents were never compelled to use a particular action consideration set. Using our experiment as an example, an agent could adopt a random policy that assigns equal probability to all available actions—thereby considering all actions while minimizing decision time. Alternatively, agents may still engage in meta-reasoning to optimize their consideration sets within their resource constraints—whether by reducing the set size, selectively retaining certain actions, or keeping the full consideration set intact while simplifying the policy itself.

Given this perspective, our framework applies equally to both cases, prescribing a notion of optimality in constructing action consideration sets contingent on policy complexity. However, we recognize that the original wording in Lines 49-50 may have inadvertently suggested a false dichotomy between meta-reasoning and resource constraints. To clarify our intended meaning, we have revised the sentence as follows:

> We propose that humans, through meta-reasoning under resource limitations, instead solve a simplified rate-distortion problem over a reduced action consideration subset.

Additionally, we appreciate the Reviewer's point that optimizing action consideration sets may compete with policy optimization for limited cognitive resources. This postulation assumes that meta-reasoning occurs online in a deliberative manner, but this need not be the case. Instead, as suggested by the general-value-based sampling heuristic, the solutions reached via meta-reasoning may be amortized over longer timescales (Binz et al., 2024), in a way that does not require deliberation during every new task encountered. We now elaborate on this idea when introducing the numerical simulations:

> In simple tasks—such as the one presented in Fig 2 and our later human experiment—it is feasible to derive reward-complexity frontiers for every possible action consideration set. However, this exhaustive approach quickly becomes impractical in more realistic settings with asymmetric reward structures and large action spaces. While an optimal action consideration set exists for any given task and policy complexity level, systematically identifying it is computationally infeasible for agents and difficult to generalize across tasks. This challenge highlights the need for heuristics that guide action consideration set formation and subsequent policy optimization—heuristics that agents could learn over longer timescales, amortize in memory, and apply flexibly to new tasks (Binz et al., 2024).
>
> Building on this motivation, we explore the advantages of biased action sampling and the interplay between policy complexity and action consideration sets by employing numerical simulations in more complex task setups. Such simulations require modeling action proposal distributions and incorporating bias-correction algorithms, both of which serve as tractable heuristics for constructing effective action consideration sets and approximating optimal policies.

Lines 115-116: I don't believe that the traditional formulation of resource rational policy compression assumes that the agent has perfect access to the ground truth $P(s)$ and $Q(s,a)$. In my experience methods that have utilized policy compression in understanding capacity-constrained decision making in humans, the source

of the $P(s)$ and $Q(s, a)$ terms comes from the experiential state probability and state-action values of an individual. These values are not supplied omnisciently from the task but estimated based on agent experience.

The original policy compression framework does not prescribe how $P(s)$ and $Q(s, a)$ are learned or estimated; rather, it merely assumes that their values are available for policy optimization via Equation 2 (Lai and Gershman, 2021; Zenon et al., 2019). Subsequent empirical studies have introduced learning and estimation components, accounting for what human agents can realistically infer within the experiment's timescale (Lai and Gershman, 2024; Gershman and Lak, 2024). To better reflect this flexibility, we have revised the sentence to avoid implying that ground-truth values of $P(s)$ and $Q(s, a)$ must be directly used:

> The above formulation assumes that the agent represents all task information: the marginal state distribution $P(s)$ and the reward structure $Q(s, a)$. However, when the action space is either large or infinite, it becomes infeasible for an agent to consider all possible actions exhaustively and represent them in a policy.

Line 133: Is this meant to suggest that $P(s)$ and $Q(s, a)$ are generally symmetric or only symmetric under the equal state probability and uniquely optimal state-action values.

We hoped to convey the latter message, and have now clarified the sentence to read:

> Given the symmetric nature of $P(s)$ and $Q(s, a)$ in this example task...

Fig 2: Is there a difference between the theoretical 'frontier corresponding to the full action space' in black and the $N_a = 6$ frontier? I don't see why both are included.

There is no difference between the two frontiers, as the full action space consists of only six actions. We included the $N_a = 6$ frontier to highlight that "considering all six available actions" is itself a decision that agents can make, just as they can choose to consider between $N_a = 1$ and 5 actions.

Line 163-164: I am having a hard time conceptualizing how or why an action would be sampled multiple times in the construction of a consideration set, as adding it to the set multiple times versus a single time seems meaningless to me. Is the importance of distinguishing between with and without replacement related to the necessity of keeping the current consideration set values in memory while sampling? I understand how repeated sampling can impact the estimate of outcomes, but in constructing consideration sets I am less clear.

We appreciate the Reviewer's thoughtful question. As noted, when sampling with replacement, repeated samples provide additional information that agents can use. For example, in the case of general-value-based sampling, the frequency with which an action is sampled can help correct for sampling biases during policy optimization—this is precisely the role of self-normalized importance sampling (SNIS). Under sampling without replacement, such bias correction would not be possible. Our interest in SNIS's bias correction stems from its demonstrated success in explaining sampling behavior in the related domain of human risky choice. To clarify this, we have expanded on our reasoning:

> In contrast to BA, a truly bias-correcting algorithm is self-normalized importance sampling (SNIS), which assumes sampling actions with replacement and is informed by the repetitive sampling of the same action (Robert et al., 2010; Owen, 2013). We consider SNIS an interesting alternative algorithm to examine, given its prior success in explaining why humans tend to oversample extreme outcomes when evaluating expected utilities of actions (Lieder et al., 2018). While sampling outcomes and actions are technically different domains, we postulated that SNIS may be an equally effective algorithm for action selection.
>
> Intuitively, any bias in the action proposal distribution would reflect in both the counts $\mathbb{I}(\alpha_j = a)$ and the denominator $P_0(a)$, such that they would cancel out in the asymptotic limit of infinite samples and thus achieve bias correction.

Additionally, because there is little empirical evidence on whether people sample actions with or without replacement, we chose not to make strong assumptions and instead simulated both possibilities. We now clarify this point in the text:

> There is limited evidence on whether humans sample actions with or without replacement, and hence we will explore both cases. Luckily, the simpler BA algorithm is compatible with both sampling methods, so we can fairly compare BA and SNIS.

Lines 271-291: The second conclusion of these simulations appears obvious, perhaps a more thorough connection to either hierarchical or continuous action space RL as suggested above can clarify why this is not the case. By this point in the manuscript I was under the impression that the main interest was in how humans handle the balance of complexity in the construction of consideration sets and the optimization of behavior under the dual constraints of information processing and limited action consideration. The final lines of this section in 288-291 further confuse the overall goal of this investigation.

We appreciate the Reviewer's feedback and recognize the need for further clarification. While the second conclusion may seem intuitive in hindsight, we do not believe it is necessarily obvious—especially for experimental researchers studying option generation. Recognizing the suboptimality requires introducing the concept of policy complexity (i.e., the state-specificity of action selection) and understanding that when policy complexity is low, considering only a few actions is not particularly detrimental—provided that the action consideration set is constructed prudently. This is because optimal policies in this regime are already concentrated on a small subset of actions.

Regarding the broader focus of the paper, we would like to clarify that it is not primarily about the competition between action consideration set size and policy complexity for limited cognitive resources, as the Reviewer suggests—though this is an interesting postulation for future research. We now incorporate it as a future direction in the Discussion:

> It would also be valuable to investigate whether action consideration set size and policy complexity compete for a shared pool of cognitive resources, or operate under separate capacity constraints analogous to the slot-continuous dichotomy in working memory (Luck and Vogel, 2013; Ma et al., 2014).

In contrast, the paper's main focus is investigating how action consideration set size and policy complexity interact synergistically to maintain near-optimal performance. Specifically, as policy complexity increases, participants should also increase the number of actions they consider. Otherwise, their policies become highly suboptimal at that level of complexity, deviating significantly from the reward-complexity optimal frontier. Conversely, when policy complexity is low, large consideration sets become less critical, allowing participants to reduce their set size to conserve cognitive resources. We now highlight this focus in the Discussion:

> The identified relationships provide a nuanced perspective on how action consideration set size and policy complexity may interact synergistically to help agents maintain near-optimal performance.

Line 302: I performed the online experiment and perhaps it was because I had read most of the paper already but I didn't find myself actively forming a consideration set. Has this environment been used in studies of human decision making in action considering set formation? Is the idea that if I was only able to remember the first three stimuli that I would only consider the first three actions and then default to the safety action for the other three? Because in that case there seems to be an effect on the state-action relationship as the agent is effectively reducing the three remaining states to a single one with a higher probability of occurring. I am not sure if I can see the direct connection between this idea of necessitating the construction of consideration sets through the use of a 'safety' action and the exact structure of the simulations as they did not have this safety action.

We appreciate the Reviewer's thoughtful reflections on the experiment. If an agent only considers the first three actions and defaults to the safety action for the remaining three, it is effectively restricting its action

consideration set to four actions instead of the full set of seven. This definition of consideration sets aligns with Morris et al. (2021), where a consideration set consists of all actions an agent actively evaluates for state-specific utility and ultimately selects from.

The observed reduction of three states to a single one is highly consistent with policy compression. Since action selection no longer differentiates between those states, the agent does not need to maintain distinct representations for them. This illustrates why adopting a high-complexity policy is costly—it requires preserving state distinctions whenever different states are mapped onto different actions (Lai and Gershman, 2021, 2024; Liu et al., 2024). However, such state abstraction is independent from the definition of action consideration sets, which concerns only the subset of actions available for selection (i.e., the policy's support).

The role of the safety action in the experiment shares key characteristics with the simulations—it is the action with the highest general value. In both cases, policy compression predicts that at low policy complexity, choice behavior is dominated by the highest-general-value action, reducing the negative impact of having a small action consideration set. We have laid out this connection when introducing the human experiment predictions:

> The latter two predictions arise from the task's reward-complexity frontier, which takes into consideration the safety action having higher general value than other actions, and that it should always be chosen at policy complexity 0 bits.

In our experiment, we designed this highest-general-value action as a "safety action" (i.e., with $Q(s, a)$ values identical across states) to preserve the symmetry of the $Q(s, a)$ matrix. This design choice simplifies the derivation of optimal frontiers for different values of N_a, as it allows us to take the maximum of two reward-complexity frontiers: one where the safety action is included in the consideration set and one where it is not (Fig 4D-F). This prevents introducing unnecessary complexity into the design while maintaining theoretical clarity.

Lines 331-333: Predictions 5 and 6 seem like they could both be caused by either smaller consideration sets alone, less complex policies alone, or a combination of these two factors. The way the experiment is constructed makes it seem hard to disentangle these two effects which I had assumed was the main interest of the manuscript. That time constraints lead to either smaller consideration sets OR less complex policies seems obvious and necessary from the way the task is structured, but what I am most interested in is how people optimize the balance between these, particularly whether they are working with a single constraint on both complexities or separate processes with independent constraints.

We acknowledge the importance of disentangling the effects of policy complexity and action consideration set size on response times (RTs). To address this, we have conducted an LME analysis using both policy complexity and N_a as simultaneous regressors (denoted LME 12):

> To assess whether the number of actions chosen contributes to RT in a way independent from its influences on policy complexity, we ran a post-hoc LME analysis and found significant positive effects for both policy complexity (fixed effects 0.375 ± 0.0510, $t(221) = 7.35$, $p < 10^{-11}$, random effects $SD = 0.0568$) and the number of actions chosen (fixed effects 0.0146 ± 0.00625, $t(221) = 2.34$, $p = 0.0200$, random effects $SD = 0.0177$). However, the statistical significance of the number of actions chosen was lost when we changed the threshold for action counting, reducing it to a trend (see Tables S2-3). Given these ambivalent results, we postulate that classical empirical results connecting the number of actions to RT may be largely explained by policy complexity, a direction that warrants future testing (Hick, 1952; Hyman, 1953).

Overall, our findings suggest that increasing the number of considered actions alone does not necessarily lead to longer RTs unless accompanied by greater policy complexity. This is intuitive: at zero policy complexity, an agent could either 1) always select the same action or 2) choose randomly from all available actions. The second strategy, despite involving a larger action consideration set, is unlikely to be inherently more time-consuming than the first.

Given this, the central message of our paper is that individuals adjust their action consideration set size to maintain near-optimality at their chosen level of policy complexity, which appears to be more strongly

constrained by time limitations. As discussed earlier, we do not take a definitive stance on whether action consideration set size and policy complexity draw from a shared cognitive resource pool or operate under separate constraints. However, this remains an intriguing question for future research.

Lines 445-449: One difficulty is the binary distinction between generally-valuable action sampling and uniform sampling. I can see that choosing between these two would be based on the complexity of the policy, but it doesn't seem like it needs to be strictly one or the other. I would imagine that there would be a way to optimize the complexity of the sampling distribution relative to a specific capacity on behavior complexity.

We thank the Reviewer for this astute comment. While there may be an optimal sampling distribution for any given task and policy complexity level, it is likely computationally infeasible to find and hard to generalize. In contrast, heuristics like general-value-based sampling may act as sufficing solutions robust across tasks, as suggested by our simulation results. We now elaborate on this idea:

> While an optimal action consideration set exists for any given task and policy complexity level, systematically identifying it is computationally infeasible for agents and difficult to generalize across tasks. This challenge highlights the need for heuristics that guide action consideration set formation and subsequent policy optimization—heuristics that agents could learn over longer timescales, amortize in memory, and apply flexibly to new tasks (Binz et al., 2024).
>
> We propose that general-value-based sampling serves as a task-general heuristic for action selection, specifically in ecologically relevant regimes of low policy complexity and small action consideration sets. By leveraging this heuristic across different tasks, agents can achieve satisficing outcomes without fully optimizing action consideration sets for each specific task (Gigerenzer and Gaissmaier, 2011).

Reviewer 2:

Overall summary

Liu & Gershman develop a theoretical framework for decision-making in situations where there are a large set of actions and consequently a large set of state-action mappings. Since humans have limited cognitive resources, they struggle to retain all state-action mappings when actions become large. As a consequence, humans make sub-optimal decisions. In situations where decisions are related to rewards, humans fail to earn the maximum amount of (average) reward in the task. The goal of this paper is to characterise this sub-optimality. In particular, the authors are interested in breaking down the sub-optimality along two (orthogonal) dimensions: "policy complexity" and number of actions in an "action consideration set size". Policy complexity is defined in information-theoretic terms but can be intuitively understood as the degree to which (learned or behaviourally exhibited) mappings between states and actions is deterministic. Action consideration set size is the number of actions that an agent chooses from. As the number of possible actions increases, this set size is characterised by a compromise made by the decision-maker, discarding some actions from the set due to limitations in cognitive capacity.

There are many things to like about the paper. The theoretical development is rigorous, it is connected with a set of predictions which are then tested in a set of experiments. The goal of the paper is ambitious but important—how to characterise human decision-making in real-world scenarios where the state-action mappings are complex and hard to learn due to the large number of possible actions.

We appreciate the Reviewer's feedback and interest in our theoretical framework. We hope to address the concerns raised.

Major comments

However, ultimately I was left quite frustrated by the paper. I am not familiar with the theoretical framework that the authors are trying to extend (though I think I understand the gist of it), so I am going to focus here on the empirical contribution of the paper and it's writing. Let me address the writing first. I feel authors can do a lot more with the way the paper is written. In it's current form, the paper is going to be comprehensible to very few people in the field. The writing style is cryptic, full of jargon and the authors have made very little effort to make it comprehensible to the average psychologist.

We appreciate the Reviewer's candid feedback and acknowledge the importance of making our manuscript accessible to a broader audience. In response to these concerns, we have revised multiple paragraphs to improve clarity and readability. In our revised manuscript, all rewritten sections are highlighted in blue. Below, we specifically outline the changes addressing the examples raised by the Reviewer. We welcome any further suggestions to enhance the clarity of our work.

1. Here is an example of a typical phrase from the paper:

> Assuming running BA on the remaining action consideration set, our predictions are supported: the general-value-based proposal distribution conferred a clear advantage at low policy complexity and small action consideration set size, compared to the uniform proposal distribution. This manifested as proximity to both the oracle proposal distribution as well as the x=0 line, which indicates closer overlap with the full-action-space reward complexity frontier and hence less suboptimality (Fig 3B Row 1).

What the authors presumably mean is that the reduction in trial-averaged reward was lower for a simulated agent that sampled actions from a general-value-based distribution compared to an agent that sampled actions from a flat distribution. The reduction in trial-averaged reward was smallest for an agent that sampled actions from an oracle distribution. Instead the authors seem to rely on a much more cryptic language and I still don't understand why the authors talk about an "overlap" with the reward-complexity frontier.

We thank the Reviewer for raising this example, and confirm that the above interpretation is correct. We have updated the paragraph to facilitate comprehension:

> Assuming no bias correction (i.e., running BA on the action consideration set, as opposed to SNIS), our predictions hold. The general-value-based proposal distribution provides a clear advantage at low policy complexity and small action consideration set sizes. Compared to the uniform proposal distribution, it yields trial-averaged rewards that are closer to those obtained with the oracle proposal distribution (Fig 3B, Row 1). Additionally, its proximity to the $x = 0$ line suggests lower suboptimality, indicating that it achieves reward levels closer to the theoretical maximum for the given policy complexity.

2. Here's another example from the section on Bias-correction algorithms:

> Notably, SNIS does not guarantee optimal performance under the current action consideration set at all policy complexity levels (whereas BA does); instead, it focuses on representing the full-action-space optimal policy at the same β without any bias, a goal whose empirical utility is subject to assessment.

Again, this strikes me as really cryptic language. Could the authors clarify why SNIS does not guarantee optimal performance under the current action consideration set. Also why is the empirical utility subject to assessment. More importantly, the authors haven't made it clear what motivates them to explore "bias-correcting" algorithms.

We apologize for the confusion here, and thank the Reviewer for raising these suggestions.

We now explict state our motivation for exploring bias-correcting algorithms like SNIS, which is informed by previous empirical works:

> We consider SNIS an interesting alternative algorithm to examine, given its prior success in explaining why humans tend to oversample extreme outcomes when evaluating expected utilities of actions (Lieder et al., 2018). While sampling outcomes and actions are technically different domains, we postulated that SNIS may be an equally effective algorithm for action selection.

We also elaborate on why SNIS does not guarantee optimal performance in terms of reward attainment, as well as the methods we use to assess its empirical utility:

> The BA and SNIS algorithms differ in their underlying objectives. BA is designed to maximize trial-averaged reward given a specific action consideration set and varying levels of policy complexity (Blahut, 1972; Arimoto, 1972). In contrast, SNIS does not aim to optimize rewards within the current consideration set but instead prioritizes unbiasedness—seeking to approximate the full-action-space optimal policy at the same β without introducing bias. Later in the paper, we assess how SNIS's objective influences reward attainment. Using an approach similar to BA in Fig 2C, we evaluate the average deviation of $\hat{\pi}^*$ (computed for each action sample $(\alpha_1, ..., \alpha_n)$) from the true task's reward-complexity frontier, across different sample sizes n. This deviation quantifies the potential loss in achievable reward.

3. Let me now turn to the empirical aspects of the paper. It is again difficult for me to understand what specific empirical phenomenon the author wanted to investigate. It occurred to me that estimating the policy complexity that a participant was using would be a very useful insight into their cognitive processes. However, it turns out that policy complexity is estimated based on previous research. This is what the authors write about estimating policy complexity (pg 20):

> Following prior work [21,24,25], we estimated the policy complexity of each participant in each ITI condition using the Hutter estimator, which computes the posterior mean value of mutual information under a symmetric Dirichlet prior [62].

Again, it would have been really useful if the authors gave some insight into how this policy complexity is estimated by this algorithm. I turned to reference [20] to gain some insight into this. This is what is stated there:

> estimation of mutual information is notoriously tricky (see Paninski, 2003). We used the Hutter estimator, which computes the posterior expected value of the mutual information under a Dirichlet prior (Hutter, 2002). We chose a symmetric Dirichlet prior with a concentration parameter $\alpha = 0.1$, which exhibits reasonably good performance when the joint distribution is sparse (Archer, Park, & Pillow, 2013).

So, not much more information there. I turned to reference [24]. This is what is stated there:

> Empirical policy complexity ... was estimated from each subject's behavior per task condition. Following [12], we use the Hutter estimator, which computes the posterior expected value of the mutual information under a symmetric Dirichlet prior [44], to estimate subjects' empirical policy complexity, or the mutual information between the observed stimuli (states) and subjects' key press responses (actions).

In other words, all the references use the same language and the reader can't understand what exactly was done to estimate the complexity. While I can infer that the authors estimated the policy complexity as the mean of a posterior (multinomial?) distribution using the Dirichlet prior, it would be really useful to have a bit more information about it. For example, in Figure 5 (the key Results figure) the policy complexity for each participant is shown as a dot. I would have liked to know what is the confidence in each point estimate. Without any further details and despite checking cited material I'm still not sure about this.

We appreciate the Reviewer's request for clarification and have now expanded on the details of the Hutter estimator in the Methods section. For further information on the estimator, we direct interested readers to the original article (Hutter, 2001):

> Following prior work (Gershman, 2020; Lai and Gershman, 2024; Liu et al., 2024), we estimated the policy complexity of each participant in each ITI condition using the Hutter estimator (Hutter, 2001). Specifically for each state, we assume a symmetric Dirichlet prior with $\alpha = 0.01$ for all actions chosen, and use the empirical action counts to reach a posterior Dirichlet distribution over action probabilities. We then estimate policy complexity as the mutual information of the posterior mean policy. The above procedure is informed by previous literature, reporting that the resulting estimates exhibit reasonably good performance when the joint distribution is sparse (Archer et al., 2014). The choice of $\alpha = 0.01$ is informed by rate-distortion theory, stating that empirical trial-averaged reward values cannot be above the reward-complexity frontier. We have chosen $\alpha = 0.01$ empirically to obey this property.

Regarding confidence intervals, we believe the Reviewer's concerns can be addressed by clarifying how individual data points are used in our analyses. Our primary focus is on identifying group-level inter-conditional changes in participant behavior as addressed by the framework. Given this focus, participant-level estimates are always aggregated to compute group-level means and confidence intervals, which serve as the foundation for hypothesis testing and statistical significance assessments in our fitted LME models.

4. The other contribution of the paper could have been to understand participants' "action consideration sets." However, this again turns out to be not one of the key contributions as the authors simply use a rough estimate for this (pg 21):

> To estimate the number of actions considered in a test block by a participant, we use the number of distinct actions chosen by that participant in that block (N_a).

How does one know that participants used the same consideration set for each decision in a block?

The contribution of this work is not on estimating action consideration sets. Rather, it focuses on exploring heuristics for sampling actions into the consideration set, and characterize interactions between consideration

sets and policy complexity that result in behavioral suboptimalities. Given this alternative focus, we decided to use a rough estimate of action consideration sets from empirical behavior, and interpret the estimate's relative change across conditions. We have summarized the paper's contributions in the Introduction section:

> By developing this computational-level framework, we seek to address several key questions: 1) Under what conditions is preferential sampling of generally valuable actions advantageous? 2) How do constraints on both policy complexity and action consideration sets interact to produce suboptimality? 3) How do humans empirically navigate such dual constraints, and do they do so in a near-optimal fashion?

We were also aware that participants may change their action consideration set halfway through a block. We tried to mitigate this possibility by giving participants training blocks to finalize their consideration sets, such that during each test block, their consideration sets hopefully remain static:

> The next three condition-specific training blocks had RT deadlines 2s, 1s, and 0.5s... These condition-specific training blocks help participants prepare themselves for the test blocks and develop their key-pressing strategy for each RT deadline.

In parallel, we also did analyses to account for instabilities in action consideration sets within a test block—counting an action as "considered" only when it is selected multiple times within the block. This addresses the situation when an action is only transiently considered:

> We re-performed all analyses based on stricter thresholds, counting a chosen action into the action consideration set size N_a if and only if the participant has chosen the action for at least two or three times within the block (see Tables S2-3). None of the conclusions change as a result of thresholding, which highlights the robustness of our findings.

5. Perhaps the real contribution of the paper is to work out the theoretical frontier in the reward-complexity space. That would be fine if one was only interested in the normative model. But it seems to me that the authors would like to make an empirical point. But when one compares participants with the theoretical frontier, there are many differences. Not only is there a cluster of participants who seem nowhere near the frontier (as the authors discuss), even the participants who are close to the frontier systematically deviate from the frontiers for middle values of policy complexity (between 0.5 and 2). Again, it would have been useful here to know what the confidence of estimated policy complexity is in each case.

We acknowledge that humans are not fully optimal—this is well-documented in prior work, where we have also outlined the theoretical frontier and observed similar levels of suboptimality (Gershman, 2020; Lai and Gershman, 2024; Liu et al., 2024). While participants do deviate from the frontier, it is worth pointing out that these deviations are relatively small compared to the full range of possible suboptimality below the frontier. In addition, is important to consider not only the black frontier (which represents the optimal frontier under the full action space) but also the N_a-specific frontiers. A key takeaway of our study is that well-documented human suboptimalities may arise because participants are effectively optimizing a simplified version of the problem with fewer actions considered, and they solved this transformed problem near-optimally. This is particularly evident for participants with $N_a = 2, 3$, and 4—while they appear below the full-action-space frontier, they are much closer to the frontiers associated with their chosen N_a.

We thank the Reviewer for pointing out empirical suboptimalities at intermediate policy complexity levels, which again occur in participants with high N_a. We postulate that these deviations may reflect noisy $Q(s, a)$ representations akin to the highly suboptimal cluster of participants identified. We now explicitly discuss the connections:

> Supporting this interpretation, we also find that even among the near-optimal participant cluster, deviations from N_a-specific frontiers tend to increase as N_a grows. This pattern suggests potential difficulties in maintaining accurate representations of additional $Q(s, a)$ entries in working memory (Luck and Vogel, 2013). Such representational noise could diminish the theoretical benefits of considering more actions as prescribed by our normative framework, providing another practical reason why agents might prefer smaller action consideration sets.

6. Of course, the authors make a number of empirical predictions (11 in all). But it turns out that most of these are not predictions from the theoretical work in this paper but from previous papers (predictions 1-6). In fact, there is nothing in the theory presented in the current paper that makes a link to RTs. And it is hard for me to see how any of these predictions are strong tests for falsifying models. I will not enumerate all of them, but in simple terms, this is what some of the predictions say:

- when response deadlines are short, participants' mapping between states and rewards will be more random (prediction 1)
- when response deadlines are short, participants will accumulate less reward (prediction 2)
- when response deadlines are short, participants are more likely to choose the safety action (prediction 3)
- when participants use a more deterministic mapping between states and actions (i.e. when participants have a better idea of which state maps to which action), they end up earning larger reward (prediction 7) and this reward becomes even higher for participants that can map more states to actions compared to participants that can map less states to action
- participants who show higher accuracy in training blocks end up being participants who are, on average, better at mapping states to actions (prediction 10).

Authors, please correct me if I'm wrong, but it seems to me that any reasonable model should make all of these predictions - so they don't seem to be predictions that will help us adjudicate between different models.

We appreciate the Reviewer's careful consideration of our empirical predictions and the opportunity to clarify their role in our study. These predictions are essential for establishing the relevance of policy compression in explaining human behavior. They also help falsify alternative accounts, which we elaborate on below.

Predictions 1 and 3: The Reviewer may be conflating policy complexity with randomness. In our experiment, when participants face shorter response deadlines, policy complexity decreases, but state-action mappings do not become more random. Instead, as predicted by Prediction 3, the policy shifts toward the action with the highest general value (the safety action in our experiment), which also reduces policy complexity because it renders action selection independent from the current state's identity. Regarding the framwork's connections to RT, we have pointed readers to our prior works:

> The first prediction arises from prior work showing that policy complexity incurs time costs (Lai and Gershman, 2024; Liu et al., 2024); the latter two predictions follow from the task's reward-complexity frontier, which considers that the safety action has a higher general value than other actions and should always be chosen at 0-bit policy complexity.

While these predictions may seem intuitive, they are not accounted for by existing computational modeling frameworks, highlighting the contribution of our theoretical approach. Notably, they are not captured by standard softmax models with adjustable noise levels, which would predict increased randomness rather than structured biases toward high-general-value actions. We have presented this line of reasoning when introducing the policy compression framework:

> However, in tasks where $P^*(a)$ is non-uniform and biased towards specific actions, then the influence of perseveration is non-trivial. This prediction differs from a traditional softmax model, where the policy approaches a uniform distribution as β approaches 0. In recent works, we identified behavioral signatures unique to policy compression—and not predicted by the traditional softmax choice rule—in human data (Lai and Gershman, 2024; Liu et al., 2024), which we will replicate in this manuscript.

Predictions 2 and 7: These predictions are not trivial consequences but arise from the policy compression framework's account of optimal cognitive resource allocation, specifically identifying policy complexity as the key constrained resource. For example, it is theoretically possible to construct policies with high complexity but low reward—such as deterministically mapping each state to a suboptimal action. However, we find that participants do not adopt such inefficient strategies. Instead, they adjust their policies in ways that maintain near-maximal reward given their chosen level of policy complexity, which aligns with the reward-complexity frontiers prescribed by the framework.

Predictions 10: This prediction extends the reasoning above by exploring how participants determine their optimal policy complexity and N_a. Our results show that participants who fail to adjust these variables based on their training performance tend to fall into the suboptimal cluster. This provides additional insight into why some participants exhibit greater suboptimality than others. We have highlighted the exploratory nature of this prediction as a venue for future process-level modeling:

> Orthogonal to the policy compression-action consideration set framework, we also made predictions regarding how a human participant decides on their policy complexity level and action consideration set size...

In summary, while many of these predictions may seem reasonable in hindsight, they are neither trivial nor universally accounted for by existing models. Their empirical confirmation supports the applicability of policy compression in describing human behavior, which further justifies our framework's joint consideration of policy complexity and action consideration sets.

7. To sum up, I think that the paper has a lot of promise and the research direction is both interesting and innovative, but in its current form the paper will be incomprehensible to many cognitive psychologists and it also fails to distinguish what it's key (empirical) contribution is.

We have elaborated on the key contributions of the study in several places:

> By developing this computational-level framework, we seek to address several key questions: 1) Under what conditions is preferential sampling of generally valuable actions advantageous? 2) How do constraints on both policy complexity and action consideration sets interact to produce suboptimality? 3) How do humans empirically navigate such dual constraints, and do they do so in a near-optimal fashion?
>
> The more important implication is the interplay between the two resource constraints. First, under partial action consideration sets, increasing policy complexity still confers greater trial-averaged reward, but also results in greater suboptimality compared to what could have been achieved under the full action space; this suboptimality can be mitigated by increasing the number of actions considered. Second, the number of actions considered provides an upper bound on policy complexity, which necessitates the increasing of both if the agent hopes to achieve greater reward.

More specifically, we have elaborated on empirical contributions—humans indeed follow normative prescriptions on how policy complexity and N_a should be changed synergistically to maintain near-optimality:

> Human data supported the framework's predictions on the influence of RT deadlines on policy complexity and the number of actions chosen, the interplay between both constraints that either enlarge or mitigate suboptimality patterns, as well as human efforts to increase both at the same time to reduce such suboptimality.

While we would love to delay some technical details to the Supporting Information, the concern is that doing so may confuse readers further. For example, readers need to minimally understand the reward-complexity frontier prescribed by policy compression, how similar analysis can be carried out for action consideration subsets, and why the framework leads to our predictions of human behavior. Otherwise, the simulations and human result sections would become incomprehensible. We have already relocated technical derivations and simulation details to the Methods section, in hope of enhancing the paper's readability.

Minor comments

Lines 167-167: After sampling actions from the proposal distribution P0(a). The simplest solution → there should be no full stop
line 177: "and hence feel that" → and hence *we feel that?
line 491: "action consideration set considerations pushes" → extra considerations

We thank the Reviewer for pointing out these typos. We have corrected them in the revised manuscript.

line 324: arise → arises

Given the sentence "the latter two predictions arise from...", we believe that "arise" is the appropriate verb.

Figure 5I: Is it possible for all complexity values to have all number of actions. If not, would a Pearson correlation be the correct measure of correlation?

The number of actions N_a places an upper bound on policy complexity ($\log_2(N_a)$ bits), but it does not constrain policy complexity from below. For example, a policy that randomly selects among all 6 actions would have 0 policy complexity, which is identical to a policy that deterministically selects one action. This flexibility allows participants to adjust policy complexity and N_a independently across task conditions (albeit in restricted ranges), making a significant Pearson correlation between them not inherently guaranteed. Given this, the observed positive correlation carries theoretical significance. We now incorporate this clarification when introducing the correlation hypothesis:

> Note that while the number of actions chosen places an upper bound on policy complexity, participants still have freedom to empirically adjust one independent from the other across task conditions (e.g., incorporate more actions while maintaining low policy complexity), such that nonsignificant correlations are in principle possible. Hence, any correlations identified in 9) may still be interpreted qualitatively with theoretical significance.

line 512: "As noise magnitude increases, we indeed see large deviations from optimality...": So can participants lie anywhere in the reward-complexity space and still be compatible with the model here? What kind of participant behaviour will falsify the model then?

The framework makes several falsifiable predictions as tested in the results. For example, if participants do not increasingly choose the safety action at low policy complexity but instead choose randomly, or do not change N_a when increasing their policy complexity, or that increases in N_a fails to reduce empirical suboptimalities at higher policy complexity, then the framework's predictions would be falsified.

In contrast, the noise magnitude investigation is an exploratory analysis aimed at understanding plausible sources of the empirical suboptimalities observed. The analysis identified noisy $Q(s, a)$ values as one possible contributor, which cannot be captured by our normative analysis assuming perfect knowledge of $Q(s, a)$. Hence, the exploratory analysis is not an attempt to rationalize deviations from the current framework, but rather a call to incorporate/tease apart their plausible sources in future studies and develop better models. This cycle of identifying deviations from optimality and incorporating additional resource formulations is common in resource-rational modeling (Lieder and Griffiths, 2020). We now clarify this interpretation:

> Given these results, it is plausible that the suboptimal cluster arises as a result of noisy Q-values, which is not captured by the normative policy compression framework.
>
> How to behaviorally separate the influence of such reward noise and policy complexity constraints merits future investigation—a deeper understanding of this distinction could help identify more realistic cognitive resource formulations.

References

Archer, E., Park, I. M., and Pillow, J. W. (2014). Bayesian entropy estimation for countable discrete distributions. *The Journal of Machine Learning Research*, 15(1):2833–2868.

Arimoto, S. (1972). An algorithm for computing the capacity of arbitrary discrete memoryless channels. *IEEE Transactions on Information Theory*, 18(1):14–20.

Binz, M., Dasgupta, I., Jagadish, A. K., Botvinick, M., Wang, J. X., and Schulz, E. (2024). Meta-learned models of cognition. *Behavioral and Brain Sciences*, 47:e147.

Blahut, R. (1972). Computation of channel capacity and rate-distortion functions. *IEEE transactions on Information Theory*, 18(4):460–473.

Del Missier, F., Visentini, M., and Mäntylä, T. (2015). Option generation in decision making: Ideation beyond memory retrieval. *Frontiers in Psychology*, 5:1584.

Gershman, S. J. (2020). Origin of perseveration in the trade-off between reward and complexity. *Cognition*, 204:104394.

Gershman, S. J. and Lak, A. (2024). Policy complexity suppresses dopamine responses. *bioRxiv*, pages 2024–09.

Gigerenzer, G. and Gaissmaier, W. (2011). Heuristic decision making. *Annual review of psychology*, 62(2011):451–482.

Hick, W. E. (1952). On the rate of gain of information. *Quarterly Journal of experimental psychology*, 4(1):11–26.

Hutter, M. (2001). Distribution of mutual information. *Advances in neural information processing systems*, 14.

Hyman, R. (1953). Stimulus information as a determinant of reaction time. *Journal of experimental psychology*, 45(3):188.

Kaiser, S., Simon, J. J., Kalis, A., Schweizer, S., Tobler, P. N., and Mojzisch, A. (2013). The cognitive and neural basis of option generation and subsequent choice. *Cognitive, Affective, & Behavioral Neuroscience*, 13:814–829.

Klein, G., Wolf, S., Militello, L., and Zsambok, C. (1995). Characteristics of skilled option generation in chess. *Organizational behavior and human decision processes*, 62(1):63–69.

Lai, L. and Gershman, S. J. (2021). Policy compression: An information bottleneck in action selection. In *Psychology of Learning and Motivation*, volume 74, pages 195–232. Elsevier.

Lai, L. and Gershman, S. J. (2024). Human decision making balances reward maximization and policy compression. *PLOS Computational Biology*, 20(4):1–32.

Leder, J., Häusser, J. A., Krumm, S., Germar, M., Schlemmer, A., Kaiser, S., Kalis, A., and Mojzisch, A. (2018). The cognitive underpinnings of option generation in everyday life decision-making: A latent variable analysis. *Cognitive Science*, 42(8):2562–2591.

Lieder, F. and Griffiths, T. L. (2020). Resource-rational analysis: Understanding human cognition as the optimal use of limited computational resources. *Behavioral and brain sciences*, 43:e1.

Lieder, F., Griffiths, T. L., and Hsu, M. (2018). Overrepresentation of extreme events in decision making reflects rational use of cognitive resources. *Psychological review*, 125(1):1.

Liu, S., Lai, L., Gershman, S. J., and Bari, B. A. (2024). Time and memory costs jointly determine a speed-accuracy trade-off and set-size effects. *PsyArXiv*.

Luck, S. J. and Vogel, E. K. (2013). Visual working memory capacity: from psychophysics and neurobiology to individual differences. *Trends in cognitive sciences*, 17(8):391–400.

Ma, W. J., Husain, M., and Bays, P. M. (2014). Changing concepts of working memory. *Nature neuroscience*, 17(3):347–356.

Morris, A., Phillips, J., Huang, K., and Cushman, F. (2021). Generating options and choosing between them depend on distinct forms of value representation. *Psychological science*, 32(11):1731–1746.

Owen, A. B. (2013). *Monte Carlo theory, methods and examples.* https://artowen.su.domains/mc/.

Raab, M., de Oliveira, R. F., and Heinen, T. (2009). How do people perceive and generate options? *Progress in brain research*, 174:49–59.

Robert, C. P., Casella, G., and Casella, G. (2010). *Introducing monte carlo methods with r*, volume 18. Springer.

Todorov, E. (2009). Efficient computation of optimal actions. *Proceedings of the national academy of sciences*, 106(28):11478–11483.

Zenon, A., Solopchuk, O., and Pezzulo, G. (2019). An information-theoretic perspective on the costs of cognition. *Neuropsychologia*, 123:5–18.
