# Supplementary material for: Action subsampling supports policy compression in large action spaces
Source: PLoS Comput Biol. 2025 Sep 5;21(9):e1013444. doi: 10.1371/journal.pcbi.1013444 (PMC12422588; doi:10.1371/journal.pcbi.1013444)
Supplement: Attachment — Submitted filename: response_to_reviewers_r2.pdf [file pcbi.1013444.s012.pdf]

Dear Dr. Doumas, Dr. Martin, and Reviewers,

Thank you very much for a second round of peer review. We have revised our manuscript according to the new set of reviewer comments. We address each Reviewer's feedback with a comment-by-comment reply. Below, we include a summary of the main revisions:

1. Experiments and Analyses
   - Introduced additional motivations and references for testing the SNIS model.
   - Clarified that the insights drawn come from the BA simulation results.
   - Conducted new analyses to reject the hypothesis that participants are selecting policy complexity levels uniformly randomly in their allowed range.
   - Corrected inconsistent terminologies, typos, and confusing sentences.
2. Figures
   - Figure 2B: explained in the figure captions why the colored frontiers end at different x-values.
   - Included a new supplementary figure (Figure S7) to support our claim that humans increase N_a and policy complexity simultaneously.

Editors:

Overall summary

Thank you for submitting your manuscript to PLOS Computational Biology. Thank you for submitting your revised manuscript. I agree with the reviewers that the revised version is much clearer than the previous version. You will see that reviewer 2 has raised a few additional points to help the clarity of the manuscript. I think addressing these points will indeed benefit the paper. Though, of course, I cannot make any guarantees, I do not foresee having to send this paper out for review again once revisions are made.

We thank the Editor for summarizing these comments, and hope to address the additional issues.

Reviewer 1:

Overall summary

Regarding my own comments, I am now much clearer on the background, motivation, and claims made in the paper thanks to the authors added clarity in the manuscript and additional connections to previous literature. The authors went line by line for all of the comments I had made in the manuscript, even minor clarification ones, which has fully addressed all my concerns. I think the current version of the paper is much stronger and I don't have any other comments or questions regarding the content. I only have one quibble below but it is fairly minor.

We thank the Reviewer for their positive feedback, and will address the remaining issue.

Minor comments

On line 103 perseveration is defined as 'state-independent actions' but to me that term is slightly different, but this isn't a big issue. But that line also references Figure 1.C which I don't think shows a state-independent policy since that's just the equation. Figure 1.D almost shows a state-independent policy but even there the probability of selecting action a1 is higher. Maybe making that distribution flat and saying (left side Figure 1.D) would be a better example of state-independent actions, or some other way.

We appreciate the Reviewer's suggestion, but we believe there may be a misunderstanding between state-independence and uniformity of the action distribution. By definition, the marginal action distribution $P(a)$ is state-independent—it defines the (marginalized) probability of selecting each action irrespective of the current state. This does not imply that $P(a)$ must assign equal probability to all actions.

In Figure 1C, the gray distribution represents the state-independent action distribution $P(a)$, which is indeed biased toward a_1. This asymmetry does not contradict its state-independence. In Figure 1D, the "low complexity" policy $\pi(a|c)$ (light green) is more similar to $P(a)$ as intended, which reflects its reduced sensitivity to the current state (with assigned codeword c).

Since I was perhaps overly familiar with the background of the paper I had not considered whether it would be easily readable by the average psychologist. These comments were raised by Reviewer 2 and I have looked through the concerns and how they are addressed. At this point it is still difficult for me to evaluate how well this has been achieved but I do agree with the author's attempts to make the paper more readable for a general audience. I also find it difficult to notice typographic mistakes like the ones raised by reviewer 2 so I will leave it to them and the editor to confirm the improvements to grammar and spelling.

We will address Reviewer 2's comments below. We thank Reviewer 1 again for their time and effort!

Reviewer 2:

Overall summary

I appreciate the changes made by the authors to improve clarity and accessibility of the manuscript. I think these changes have been positive and have made me appreciate their work a bit more. While the manuscript is definitely in a better shape, there are a few remaining concerns that, in my view, should be addressed.

We appreciate the Reviewer's feedback and will address the concerns raised.

Major comments

1. The sections that I still found difficult to parse is the one on 'Bias-correction algorithm' (pg 7). The goal here seems to be comparing the Blahut-Arimoto algorithm with an algorithm that does not produce a biased estimate. The authors haven't spent much time in the manuscript motivating this goal, i.e. discussing how much of a problem generating a biased estimate could be. It strikes me that the manuscript could benefit from simulations showing how a bias in $P_0(a)$ affects the results of the Blahut-Arimoto algorithm before considering alternative models (if alternative models are to be presented)

We have simulated the effected of biased $P_0(a)$ distributions on the Blahut-Arimoto algorithm in Figures 3 and S1-5. This has led to our conclusion that a $P_0(a)$ distribution biased by general action value confers advantages at low policy complexity and small N_a.

Moreover, the alternative model (SNIS), which produces an unbiased estimate, seems like a strawman (or, at least, an afterthought). Unsurprisingly, this model is rejected a few pages later (pg 9-10). Furthermore, SNIS only produces an unbiased estimate "in the asymptotic limit of infinite samples". So, it's not really a cognitively plausible model. If SNIS is not critical to the author's thesis, perhaps a better place for it will be the Appendix. That will further increase the accessibility of the manuscript.

We appreciate the Reviewer's concern and the opportunity to clarify the motivation behind including SNIS in the main text. We believe SNIS is a theoretically relevant alternative, given its established use in modeling human cognition and its close ties to resource-rational sampling frameworks. These connections make it a natural candidate for comparison in our investigation of action sampling. In fact, SNIS was the first algorithm we considered prior to developing the BA model with no bias correction. To better communicate this rationale, we have added references and expanded our explanation as follows:

> We consider SNIS a compelling alternative algorithm to examine for two main reasons. First, SNIS has previously been shown to account for the human tendency to oversample extreme outcomes when estimating the expected utilities of actions (Lieder et al., 2018). Second, similar importance sampling algorithms have successfully explained a wide range of human behaviors under resource constraints, including multiple object tracking (Vul et al., 2009), concept learning (Thaker et al., 2017), reinforcement learning (Daw and Courville, 2008), and sentence parsing (Levy et al., 2008). While sampling outcomes and actions are technically different domains, we postulated that SNIS may be an equally effective algorithm for action selection.

We would also like to clarify that SNIS is cognitively plausible. While its asymptotic unbiasedness is a desirable property in the limit of infinite samples, the algorithm can also be applied with a finite number of samples—consistent with bounded rationality principles commonly invoked in cognitive modeling. This is precisely how SNIS and related sampling algorithms have been used in the studies cited above, as well as our current work.

2. Related to point 1, the manuscript doesn't spend much time discussing the results of SNIS algorithm in Figure 3B. These results, at least at first glance, seem strange. While the BA algorithm (top row) predicts that an increase in policy complexity leads to better trial-average reward – which makes sense – SNIS predicts a non-monotonic relationship between policy complexity and reward. That is, sometimes

increasing complexity leads to better trial average reward, while other times it leads to worse trial average reward. Similarly, the comparison between proposal distributions leads to to some odd results here. In the case of the BA algorithm sampling from the oracle distribution performs better than the value-based action sampling, which performs better than uniform action sampling. This makes sense. But for SNIS, there is no clear relationship. For some policy complexity values the value-based sampling performs better than uniform action sampling, while for other values the relationship reverses. Moreover, for some policy complexity values, both value-based and uniform sampling perform /better/ than the oracle distribution. I don't understand why this is the case and I can't see an explanation in the manuscript for these effects. If the authors think all this does not need to be discussed, then this again speaks for moving SNIS to the appendix.

We appreciate the Reviewer's close reading of Figure 3B and agree that the SNIS results are less straightforward than those of the BA model. Our decision to focus primarily on the BA algorithm stems from its superior performance and empirical consistency with human behavior. That being said, we believe SNIS remains important to include in the main text—not only because of its relevance in the literature (as noted in our previous response), but also because it serves as a cautionary example regarding the uncritical application of statistical approximation algorithms to model cognition. We have emphasized this point in the Discussion:

> The above finding provides a cautionary example of applying statistical approximation algorithms to understanding human behavior (Gershman et al., 2012; Dasgupta et al., 2017; Lieder et al., 2018). Given the resource constraints that humans face, asymptotic optimality may not be enough to motivate the usage of a particular algorithm in naturalistic settings, and a case-by-case assessment of their performance in low sample-complexity regimes may be necessary (Vul et al., 2014).

We now turn to the Reviewer's observation regarding the lack of consistent trends in the SNIS results. The absence of a clear advantage for the oracle or general-value-based proposal distributions across tasks stems from SNIS's bias-correction mechanism, which reweighs the sampled action counts through its proposal distribution denominator (see Equation 3). This correction can be more or less effective depending on the specific reward structure of the task, leading to variable performance. While it would indeed be valuable to systematically investigate the task conditions under which SNIS performs well, such a technical analysis falls outside the scope of the present work and is better suited for future research.

3. Pearson correlation: I'm afraid I don't buy the author's defence of using Pearson's r to measure the correlation between number of actions chosen and policy complexity, in Figure 5I (see my comments and author's response). I understand that the authors are arguing that it is theoretically possible to have zero correlation and therefore having a correlation of 0.67 is significant. But is this a strong or a weak correlation? The authors suggest that this correlation is "positive and strong" (line 442). But I'm not so sure.

Here's how I'm thinking about it: When number of actions are 2, the policy complexity can only vary from 0 to log(2). When number of actions are 6, the policy complexity can vary between 0 and log(6). That is, the range increases as number of actions increase. Because of this, if an agent chose the policy complexity completely randomly from a uniform distribution you will get a correlation between number of actions and policy complexity. So, is a correlation of 0.67 "positive and strong"n? Well, firstly correlation in this case cannot be negative. Also, the correct baseline for the correlation here is not 0. Rather, it is the correlation for this agent who chooses complexities randomly from a uniform distribution. Can the authors compute this before claiming that the correlation is strong?

We thank the Reviewer for raising this thoughtful alternative explanation. In response, we conducted additional analyses and included a new supplementary figure (Figure S7) to directly address this point:

> One potential concern is that the number of actions chosen (N_a) places an upper bound on policy complexity ($\log_2(N_a)$ bits), which raises the alternative explanation that the observed correlation simply reflects a uniform sampling of policy complexity within its allowable range.

To address this, we performed post hoc Kolmogorov-Smirnov tests on the empirical distribution of policy complexity at each N_a. These tests revealed significant deviations from uniformity for $N_a = 2, 3, 6, 7$ ($p < 10^{-4}$). Moreover, visual inspection of the policy complexity distributions revealed a shift from right-skewness to left-skewness as N_a increased from 2 to 6 (Fig S7). This pattern may explain the inability to reject uniformity for intermediate N_a values ($N_a = 4, 5$), and supports the prediction that humans tend to increase both N_a and policy complexity across RT deadline conditions in an effort to reduce suboptimality.

Minor comments

Figure 2B: Could the authors make it clear in the caption (or where they discuss this figure) that policy complexity can only vary between 0 and log(Na). That is why the curves in this figure have different ranges. I remember this was one of my key confusions when I first saw this figure.

We agree with the Reviewer and now include relevant information in the Figure 2 captions:

> Note that the maximum policy complexity allowed by N_a is $\log_2(N_a)$ bits, such that different colored curves end at different policy complexity levels.

Figure 2B: The frontier for the full action space is the same as the frontier for Na=6 because there are only six actions in the full action space. Therefore, the green lines and black lines overlap, by definition. So, why plot both these lines on the same graph. Wouldn't it be better to remove the black line and change the legend "Na=6" to "Na=6 (full)" or something similar?

We appreciate the Reviewer's observation, but believe it is still important to distinguish between the two lines. The black line reflects the theoretical reward frontier achievable when using the full action space, which in our implementation consists of seven actions—not six. While using $N_a = 6$ happens to yield the same frontier in this particular task, this is not guaranteed in general. In many cases, relying on a subset of available actions is insufficient for reaching optimal performance. To highlight this conceptual distinction, we have retained the black line as a reference for the true upper bound under the full action space.

line 161: "Following the two-step framework", I think this was called the "two-stage model of open-ended decision-making" above. Worth maintaining the same language so as not to confuse the reader. Also, I would suggest that the authors insert a reference to Figure 1A again here to make it clear that they are talking about a concept previously developed in the manuscript.

We thank the Reviewer for raising this terminology inconsistency. We have updated the sentence to read:

> Following the two-stage decision-making architecture described earlier (Fig 1A) (Phillips et al., 2019), we first consider the proposal distribution $P_0(a)$ through which actions are sampled and enter the consideration set.

line 174: "orthogonal dimensio": Did the authors mean "another model-parameter worth exploring"? This is also the first time the authors mention comparing the biased vs unbiased estimators. But it hasn't been motivated (see point 2 above).

We have incorporated the Reviewer's proposed terminology. The sentence now reads:

> Another model parameter worth exploring is whether actions are sampled with or without replacement from these proposal distributions.

Regarding our motivation to test both biased and unbiased estimators, please see our response to Point 2 above, which focuses on SNIS's relevance to sampling-based cognitive theories and its successful applications to previous empirical studies.

line 181: “Notably BA is agnostic towards the number of times a particular action is sampled”. What does it mean for an algorithm to be agnostic? Do you mean that, in principle, BA can be set up in a way that it samples actions with replacement? Again, there is a lot in the manuscript that can be improved with the discussion of bias in BA algorithm.

We apologize for the confusion. We have updated the sentence to read:

> Notably, BA is indifferent to how often each action is sampled—it considers only the number of distinct actions in the sample, without accounting for their frequencies. Furthermore, BA does not correct for any bias in the $P_0(a)$ that may make some actions more likely to be sampled than others.

We have also elaborated on our reasoning for why BA remains compatible with sampling actions with replacement:

> Luckily, the simpler BA algorithm is compatible with both sampling methods—when sampling actions with replacement, it simply does not make use of how often each action is sampled, relying only on the set of unique actions. Hence, we can fairly compare BA and SNIS in the regime of sampling with replacement.

line 296: “the suboptimality in trial-averaged reward increases with policy complexity” Did the authors mean for the BA algorithm? This does not seem to be the case for SNIS.

We confirm that the Reviewer's interpretation is correct. We have rewritten previous sentences to clarify that our subsequent conclusions arise from the BA simulation results:

> This allows us to extract general insight on the interaction between the two sources of cognitive constraints—policy complexity and action consideration set size—based on the BA simulation results.

line 179: “sample.The” missing space.
line 260: “x=0” Did the authors mean y=0 line?
line 335 (and throughout this paragraph): Fig A-B should be Fig 4A-B, similarly for Fig C-E.
“The first prediction arise” → arises

We thank the Reviewer for identifying these typos. We have now corrected them.

References

Dasgupta, I., Schulz, E., and Gershman, S. J. (2017). Where do hypotheses come from? *Cognitive psychology*, 96:1–25.

Daw, N. and Courville, A. (2008). The pigeon as particle filter. *Advances in neural information processing systems*, 20:369–376.

Gershman, S. J., Vul, E., and Tenenbaum, J. B. (2012). Multistability and perceptual inference. *Neural Computation*, 24(1):1–24.

Levy, R., Reali, F., and Griffiths, T. (2008). Modeling the effects of memory on human online sentence processing with particle filters. *Advances in neural information processing systems*, 21.

Lieder, F., Griffiths, T. L., and Hsu, M. (2018). Overrepresentation of extreme events in decision making reflects rational use of cognitive resources. *Psychological review*, 125(1):1.

Phillips, J., Morris, A., and Cushman, F. (2019). How we know what not to think. *Trends in cognitive sciences*, 23(12):1026–1040.

Thaker, P., Tenenbaum, J. B., and Gershman, S. J. (2017). Online learning of symbolic concepts. *Journal of Mathematical Psychology*, 77:10–20.

Vul, E., Alvarez, G., Tenenbaum, J., and Black, M. (2009). Explaining human multiple object tracking as resource-constrained approximate inference in a dynamic probabilistic model. *Advances in neural information processing systems*, 22.

Vul, E., Goodman, N., Griffiths, T. L., and Tenenbaum, J. B. (2014). One and done? optimal decisions from very few samples. *Cognitive science*, 38(4):599–637.
